# Carbohydrate scaffolds as glycosyltransferase inhibitors with *in vivo* antibacterial activity

**DOI:** 10.1038/ncomms8719

**Published:** 2015-07-21

**Authors:** Johannes Zuegg, Craig Muldoon, George Adamson, Declan McKeveney, Giang Le Thanh, Rajaratnam Premraj, Bernd Becker, Mu Cheng, Alysha G. Elliott, Johnny X. Huang, Mark S. Butler, Megha Bajaj, Joachim Seifert, Latika Singh, Nicola F. Galley, David I. Roper, Adrian J. Lloyd, Christopher G. Dowson, Ting-Jen Cheng, Wei-Chieh Cheng, Dieter Demon, Evelyne Meyer, Wim Meutermans, Matthew A. Cooper

**Affiliations:** 1 Institute for Molecular Bioscience, The University of Queensland, St Lucia, Queensland 4072, Australia; 2 Alchemia Ltd, PO Box 4851, Eight Mile Plains, Brisbane, Queensland 4113, Australia; 3 School of Life Science, University of Warwick, Gibbet Hill Road, Coventry CV4 7AL, UK; 4 Genomics Research Center, Academia Sinica, 128 Academia Road, Section 2, Taipei 115, Taiwan; 5 Faculty of Veterinary Medicine, Laboratory of Biochemistry, Ghent University, Salisburylaan 133, 9820 Merelbeke, Belgium

## Abstract

The rapid rise of multi-drug-resistant bacteria is a global healthcare crisis, and new antibiotics are urgently required, especially those with modes of action that have low-resistance potential. One promising lead is the liposaccharide antibiotic moenomycin that inhibits bacterial glycosyltransferases, which are essential for peptidoglycan polymerization, while displaying a low rate of resistance. Unfortunately, the lipophilicity of moenomycin leads to unfavourable pharmacokinetic properties that render it unsuitable for systemic administration. In this study, we show that using moenomycin and other glycosyltransferase inhibitors as templates, we were able to synthesize compound libraries based on novel pyranose scaffold chemistry, with moenomycin-like activity, but with improved drug-like properties. The novel compounds exhibit *in vitro* inhibition comparable to moenomycin, with low toxicity and good efficacy in several *in vivo* models of infection. This approach based on non-planar carbohydrate scaffolds provides a new opportunity to develop new antibiotics with low propensity for resistance induction.

## Abstract (web summary)

The inhibition of bacterial glycosyltransferase has the potential to be an effective therapeutic target against drug resistance bacteria. Here, the authors present a novel class of inhibitor compounds based on a monosaccharide scaffold, which are able to eliminate bacterial infections in mice.

## Graphical abstract

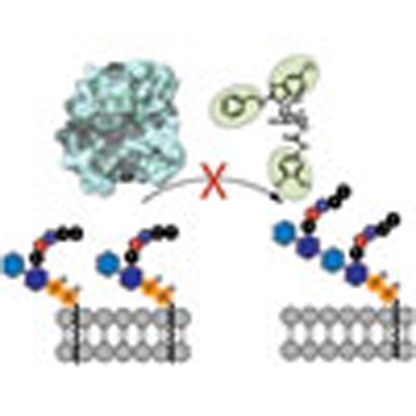

Peptidoglycan glycosyltransferases (GT) and transpeptidases (TP) are two key enzymes in the final steps of peptidoglycan (PG) biosynthesis essential for bacterial cell wall integrity and stability. GTs catalyse the polymerization of lipid II disaccharide units, forming a long chain of alternating β-1,4-linked *N*-acetylglucosamines and *N*-acetylmuramic acid, leading to a linear glycan chain and the release of undecaprenyl-pyrophosphate carrier^1^,^2^,^3^. These carbohydrate chains are further crosslinked by TP enzymes, forming linkages between the peptide chain and the D-alanine of a neighboring unit (Fig. 1). GT and TP enzymes are unique to bacteria and are expressed either as individual domains, monofunctional GT (MGT) and penicillin-binding proteins (PBP), respectively or as bifunctional proteins that possess both GT and TP domains (class A PBP)^4^,^5^.

Inhibition of extracellular bacterial cell wall synthesis has been a very successful strategy in the development of many important antibacterial agents, with teixobactin^6^, one of the most recently reported. The β-lactam class, which includes cephalosporins, monobactams and carbapenems, inhibit PG crosslinking by covalently binding to the TP enzyme, while glycopeptides such as vancomycin bind directly to the lipid II unit and sterically inhibit further polymerization and crosslinking of PG. Bacteria developed resistance to cell wall inhibitors via β-lactamases, thickened cell walls and modification of the lipid II unit, with extended-spectrum β-lactamases such as NDM-1 and vancomycin-resistant enterococci representing a significant health threat^7^. Glycolipopeptides (for example, ramoplanin), cyclic peptides (for example, AC98-6446) and lantibiotics (for example, nisin or NVB302) also bind to lipid II of Gram-positive bacteria^8^. Only nisin has reached the market, and then only as a food preservative^9^.

Antibacterial compounds that bind directly to GT have never been developed for human use. Of the few examples reported in the literature, moenomycin is by far the best described^10^. Moenomycin is produced by various streptomyces species and has a broad-spectrum activity against a range of Gram-positive bacteria. The poor pharmacokinetic properties of moenomycin have prevented further clinical development^10^,^11^, and it has been commercialized only as a ‘growth promoter' within animal feed stocks (Flavomycin and Flavophospholipol). Despite evidence that regular application of antibiotics as growth promoters in animals in general leads to increased antibiotic resistance^12^,^13^, remarkably no moenomycin-resistant bacteria in animals have been reported to date^14^,^15^. *In vitro* resistance induction experiments showed extremely slow development of resistance with low-resistant frequencies^16^, as well as no transferable resistance between organisms^17^,^18^, no cross-resistance to other antimicrobials or co-selection of resistant strains^19^. Intrinsic resistance in moenomycin-producing organisms is not associated with the biosynthesis cluster, but is likely to arise from the presence of GT's with low affinity for moenomycin, or some peculiarities of their cell wall organization^10^. Further, moenomycin is primarily accumulated inside of the cells, while its target is located on the cell surface^20^,^21^. *In vitro*-induced resistance with *S. aureus* showed mutations in the binding site of PBP2 with reduced affinity for moenomycin as well as its ligand, resulting in strains with shorter PG polymers and major cell division defects^16^. The lack of a specific resistance mechanism and the paucity of antibiotics that specifically mimic the carbohydrate portion of bacterial lipid II suggest that direct GT inhibition remains an attractive strategy for the development of novel antibacterial agents with low potential for resistance development.

Moenomycin A is a highly functionalized pentasaccharide attached via a phosphoglycerate linkage to a polyprenyl chain^22^ (Fig. 1) that binds competitively to GT enzymes by mimicking the disaccharide–pyrophosphate–prenol linkage of the donor lipid II^10^,^23^,^24^. Although the E and F rings and the phosphoglycerate (G) portion of moenomycin A are important for GT inhibitory activity, analogues of this pharmacophore subunit did not maintain whole cell antibacterial activity^25^,^26^. Attempts to mimic the EFG structural fragment with disaccharide derivatives^27^,^28^,^29^,^30^ resulted in compounds (such as TS30153 (ref. 17)) with cell-based activity, that is, minimum inhibitory concentration (MIC) of 3.12 and 12.5 μg ml^−1^ against staphylococci and enterococci, but with no *in vivo* activity. Compound TS30153 (ref. 17) has three hydrophobic binding elements that mimic the acyl and alkyloxy moieties of moenomycin A (Fig. 2b). Attempts to mimic directly lipid II^31^, or the ring F of moenomycin^32^, with monosaccharide scaffolds gave compounds with only low to medium activity (MIC=60 μM against *Bacillus cereus*^31^). More recently, *de novo* inhibitors for GT discovered using high-throughput screening^33^,^34^,^35^ or *in silico* methods^36^, were shown to have improved *in vitro* activity (MIC=0.25 μM against MRSA^33^), but no *in vivo* activity.

In this study, we explore novel chemistry based on a monosaccharide scaffold^37^ to mimic the essential structure features of moenomycin and to improve the drug-like properties, in particular reduced molecular weight and hydrophobicity. Compared with other scaffolds, the monosaccharide scaffold approach provides structural diversity using up to five chiral attachment points within a small volume^38^. This allows for more efficient pharmacophore optimization, while still enabling the generation of a broad structural diversity to scope and improve activity and physicochemical properties. Here we use the approach to produce moenomycin-focused libraries and select compounds with *in vitro* antibacterial activity and *in silico* potential to inhibit the GT enzyme. We demonstrate the strength of this strategy with two of the most promising candidates showing inhibition of GT and PG synthesis in *in vitro* assays, as well as *in vivo* efficacy in eliminating *S. aureus* infection from a mouse mammary gland.

## Results

### Design and synthesis

**Figure 1:**
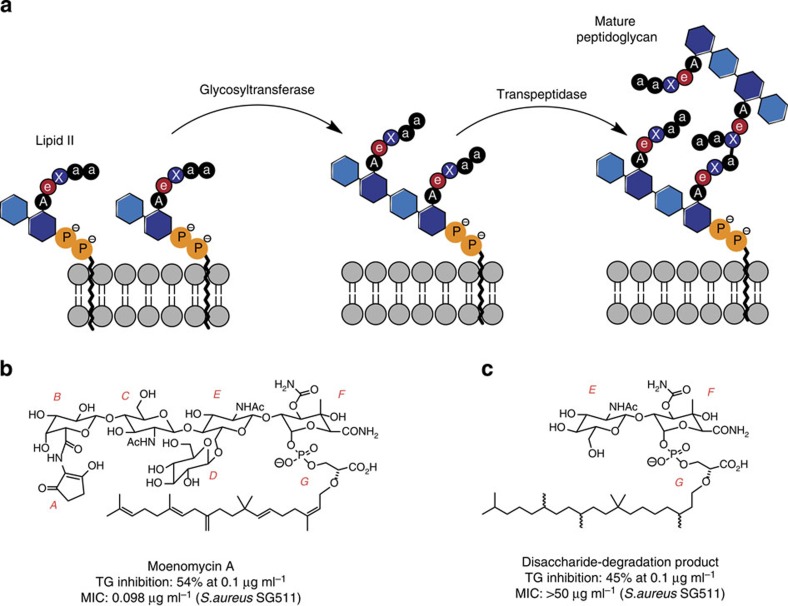
Overview of PG cell wall synthesis and inhibitors. (**a**) PG synthesis in bacteria from lipid II with subsequent GT and TP catalysis, with A: L-Ala, a: D-Ala, e: D-iGln, X: either D-Lys(Ala_5_) in case of *Staphylococcus*, or mDap in case of *Bacillus*. (**b**) Structure and *in vitro* activity of moenomycin A, indicating the different moieties with A to G. (**c**) Structure and *in vitro* activity of moenomycin's disaccharide degradation product.

**Figure 2:**
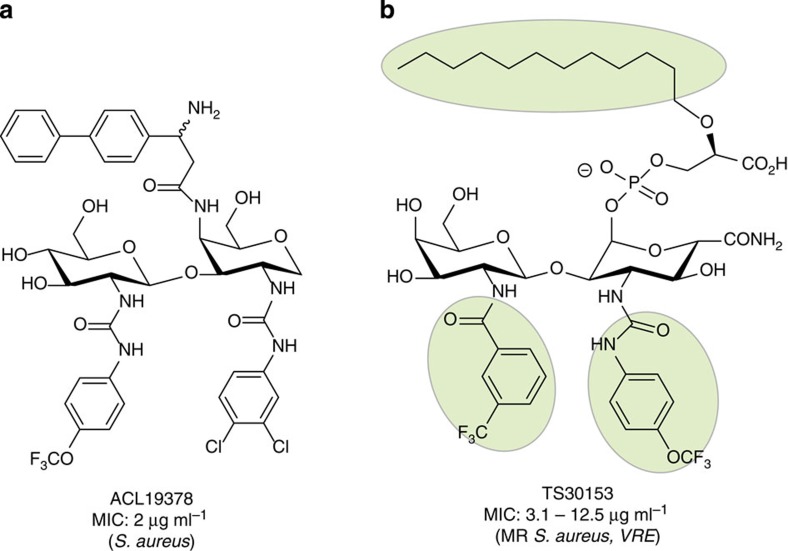
Disaccharide templates for design of monosaccharides. (**a**) ACL19378, representative compounds from disaccharide library. (**b**) TS30153 (ref. 27) highlighting the three binding elements required for GT inhibition in green.

**Figure 3:**
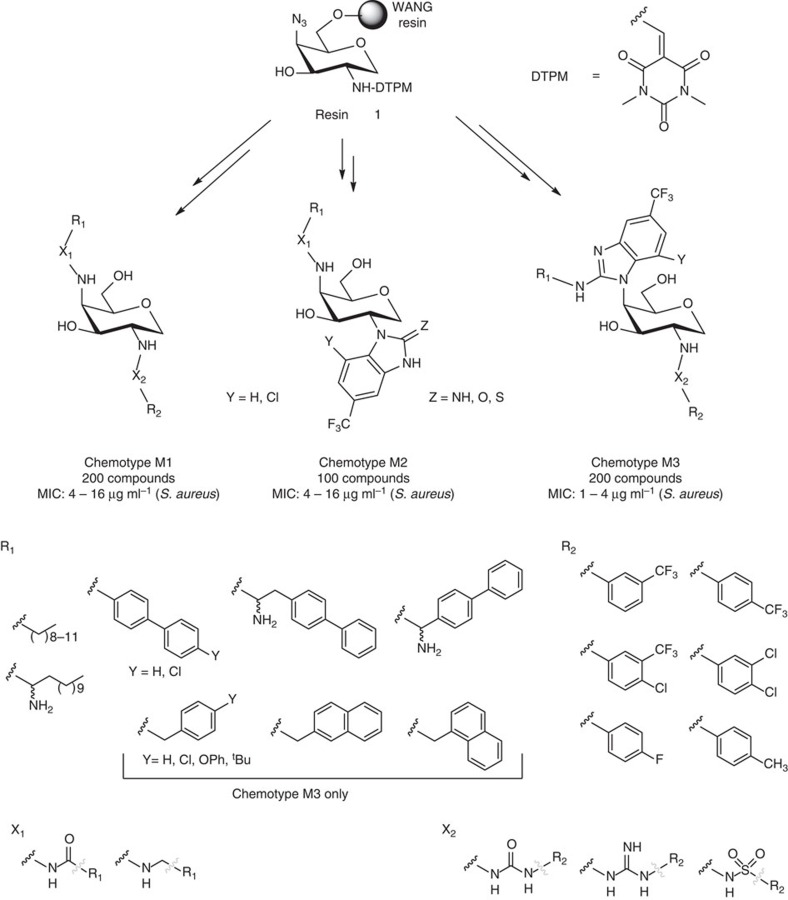
Design of monosaccharide libraries. The figure illustrates the common starting building block, the three different chemotypes (M1, M2 and M3) and corresponding diversification at each substitution point.

We synthesized a small library of compounds by replacing the phosphoglycerate/phosphate moieties (G, Fig. 1) with simpler lipophilic substituents (that is, phenyl, biphenyl or naphthyl groups linked via a urea) and changing the orientation and nature of ring F. This approach yielded compounds like ACL19378 (Fig. 2a, Supplementary Figs 1–8) and ACL19333 (Supplementary Fig. 1), with MICs against Gram-positive bacteria in the range of 2 μg ml^−1^, but with limited activity in the presence of 50% serum. In the second stage, we used the disaccharide structure–activity relationship information to design more synthetically feasible and smaller monosaccharide molecules. A versatile solid-phase method was developed to rapidly synthesize the representatives of three different core chemotypes M1 to M3, starting from a single monosaccharide building block, that is, 1,5-anhydro-galactitol (see Fig. 3). Chemotype M1 explored the option of using only two of the hydrophobic elements, whereas the other two, M2 and M3, used a benzimidazole moiety as the third hydrophobic group (Fig. 3).

**Figure 4:**
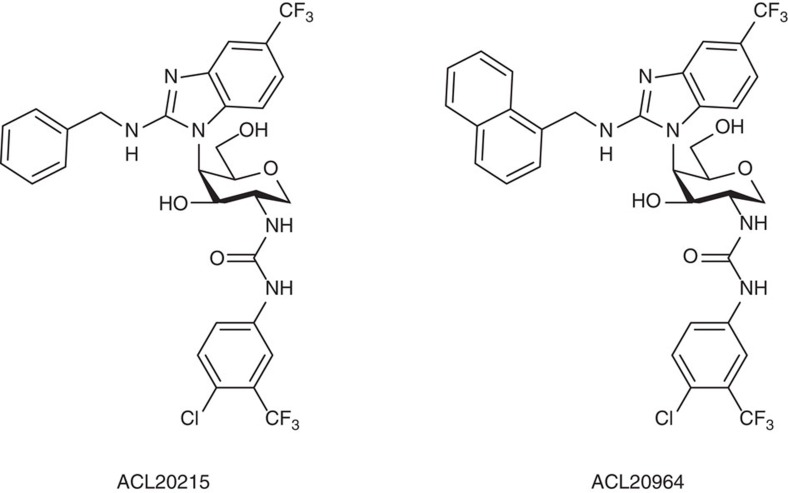
Structures of ACL20215 and ACL20965. ACL20215 and ACL20965 are two of the most active monosaccharide compounds.

Five hundred compounds were thus synthesized combinatorially on solid-phase resin, purified using high-performance liquid chromatography, and tested for their MIC activity against two Gram-positive staphylococcal strains (methicillin-sensitive (MSSA) and methicillin-resistant *S. aureus* (MRSA)), three enterococcal strains and *Escherichia coli* as a Gram-negative control. Although most compounds were inactive against *E. coli*, many compounds displayed activity against the Gram-positive strains. The derivatives with Gram-positive antibacterial activity generally contained a lipophilic substituent such as an alkyl moiety (minimum length of 10 carbon atoms) or a biaryl, and one or two electron-deficient aryl groups. All active compounds were then tested for haemolytic activity and, after filtering out the haemolytic compounds, a series of compounds of chemotype M3 containing substituted amino-benzimidazoles were selected for further study (Supplementary Table 1). Two compounds (Fig. 4), ACL20215 (Supplementary Figs 10–15) and ACL20964 (Supplementary Figs 16–20), showed broad activity against a range of drug resistant *S. aureus* strains, including MRSA, GISA (glycopeptide-intermediate *S. aureus*), VRSA (vancomycin-resistant *S. aureus*) and DRSA (daptomycin-resistant *S. aureus*) and multi-drug-resistant *S. pneumoniae*, with low haemolytic activity against human red blood cells (Table 1). ACL20215 was assayed for resistance potential and showed a spontaneous mutation frequency against *S. aureus* (ATCC 13709; Smith strain) of less than 2.5 × 10^−10^ at four times its MIC value.

### Evaluation of mode of action

**Figure 5:**
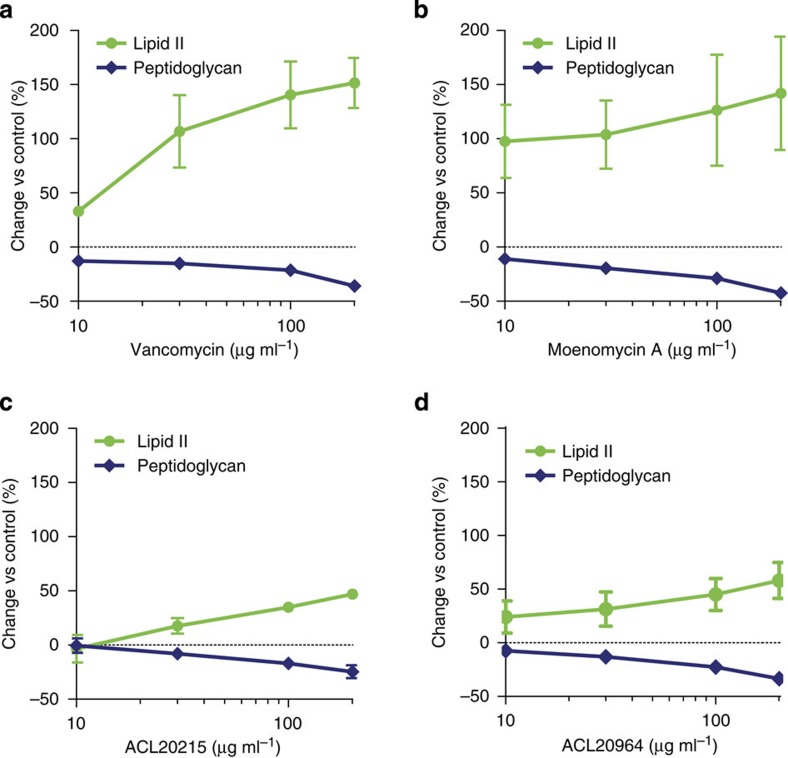
Inhibition of PG synthesis. The inhibitory effect in bacterial PG biosynthesis within a crude *B. megaterium* membrane is shown for (**a**) vancomycin, (**b**) moenomycin A, (**c**) ACL20215 and (**d**) ACL20964, showing the relative change of lipid II and PG isolated from the crude membrane by TLC, after 3 h, compared with non-antibiotic treatment. Error bars show s.d. for *n*=3.

To elucidate the mode of action of the inhibitors, we conducted various GT enzyme inhibition assays. We first examined the inhibitory effect of the compounds in a bacterial membrane environment, comparing ACL20215 and ACL20964 in an *in vitro* assay for bacterial PG biosynthesis, using crude *Bacillus megaterium* membrane preparations. This assay monitors [^14^C]UDP-GlcNAc incorporation into lipid II and mature PG, in the presence of different concentrations of antibiotics^39^. Owing to the sequential nature of the glycosyltransfer and transpeptidation, it is not possible to determine at which step PG biosynthesis is blocked in this assay. The low concentration of lipid II can be detected by thin-layer chromatography (TLC) separation and subsequent phosphorimaging of pre-solubilized membranes, which was incubated with the cytoplasmic PG precursors UDP-MurNAc-pentapeptide and UDP-[^14^C]GlcNAc^41^,^42^. As shown in Fig. 5 (and Supplementary Table 2 and Fig. 21), both ACL20215 and ACL20964 caused an inhibition of mature PG production at a concentration of 200 μg ml^−1^, to a similar extent as the controls, vancomycin and moenomycin A. Both inhibitors also caused an accumulation of lipid II, although to a lesser extent compared with vancomycin or moenomycin A.

Membrane-disruption experiments were performed using *S. aureus* (ATCC 25923) in combination with membrane potential-sensitive cyanine dye diSC_3_5 (ref. 43). Neither compounds showed membrane disruption (Supplementary Fig. 22) compared with a positive control Citropin 1.1 (refs 44, 45, 46), which suggested that the GT inhibitors disrupt PG biosynthesis without disrupting the cell membrane.

**Figure 6:**
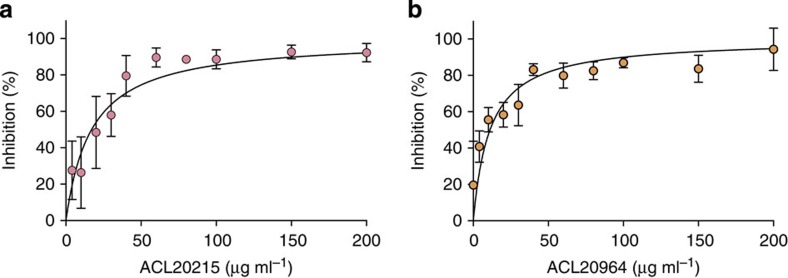
Inhibition of glycosyltransferase. The inhibition of MGT from *S. aureus* is shown for ACL20215 (**a**) and ACL20964 (**b**), by measuring the transformation rate of fluorescent lipid II analogue and comparing it with the maximum inhibitory effect of moenomycin A at 50 μM or 79.2 μg ml^−1^. Error bars show s.d. for *n*=4.

We then monitored the transformation of fluorescent NBD-lipid II by two different GT enzymes, PBP1 from *Clostridium difficile* and MGT from *S. aureus*. The single concentration test at 200 μg ml^−1^ revealed that both inhibitors showed an inhibitory effect against MGT *S. aureus*, while showing only moderate effect against PBP1 *C. difficile* (Supplementary Fig. 23). Confirmative dose–response assays were subsequently conducted with continuous fluorescent activity assay using a dansyl-labelled, lysine-lipid II substrate^47^. The assay revealed that both ACL20215 and ACL20964 were able to inhibit MGT from *S. aureus* with an IC_50_ of 17 and 11 μg ml^−1^, respectively (Fig. 6). In the same assay, moenomycin A was used as positive control showing an IC_50_ of 5 μM or 8 μg ml^−1^.

### Virtual docking

**Figure 7:**
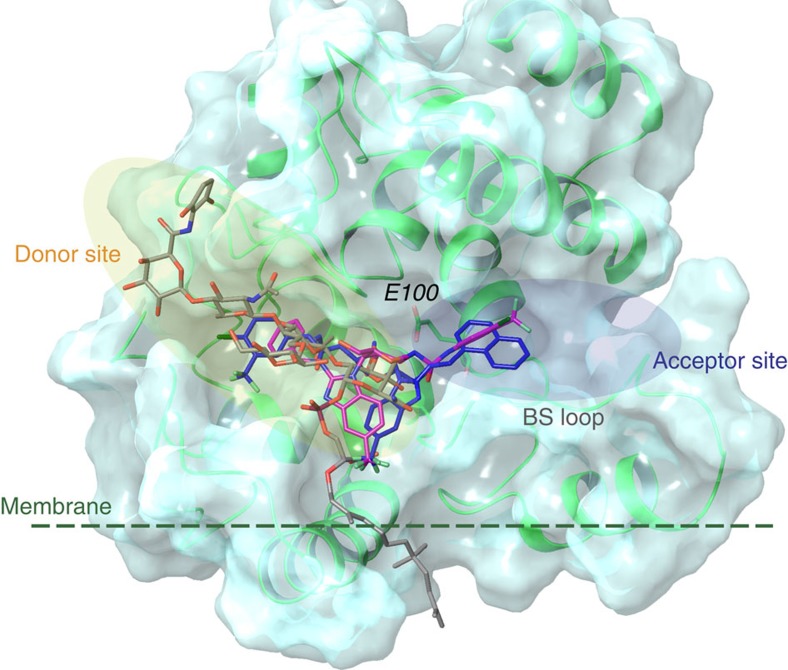
Virtual docking studies of ACL20215 and ACL20215. The virtual docking poses are shown for ACL20215 (pink) and ACL20964 (blue) within the GT domain of MGT *S. aureus* (pdb: 3HZS^48^) shown as ribbon (green) and surface representation. Binding orientation of moenomycin is shown as found in the corresponding crystal structure (grey). The structure illustrates that both inhibitors are able to occupy the donor, as well as part of the acceptor sites. The dotted line is illustrating the suggested membrane interface^49^,^50^. BS loop marks the region of the binding site loop with high conformational variation or disorder in the different crystal structure, while E100 marks the active site residue Glu_100_.

Several crystal structures of GT domains have been reported for Gram-positive (MGT^48^,^49^ and PBP2 (refs 50, 51) from *S. aureus*) and Gram-negative bacteria (PBP1 (ref. 52) from *E. coli* and PGT^11^,^53^ from *Aquifex aeolicus*), showing a high structural similarity between the difference species. One main feature of the structures is a binding site loop (MGT *S. aureus* Phe_120_–Gly_130_; PBP2 *S. aureus* Gly_134_–Gly_145_)^54^ located between the donor-binding site, occupied by moenomycin, and the acceptor binding site occupied by the incoming lipid II molecule. This binding site loop is highly flexibly and partly disordered in most of the crystal structures (see Supplementary Table 3). Even when the loop is resolved, it can occupy different conformations, either separating the donor from the acceptor sites or opening a groove between the sites (see Supplementary Fig. 24 and Supplementary Note 1). We have carried out *in silico* virtual docking with the monosaccharides ACL20215 and ACL20964, choosing the receptor model (and loop conformation), which best reproduced the binding orientation of moenomycin. A receptor model based on the crystal structure of MGT from *S. aureus* with a loop conformation blocking the access to the acceptor site, 3HZS^48^, was thereby selected (see Supplementary Fig. 25). As shown in Fig. 7, the benzimidazole group of both inhibitors was located similarly to portion G of moenomycin, with one of the other hydrophobic groups located in the donor-binding site (similar to ring E of moenomycin), and the other was located towards the acceptor site not occupied by moenomycin. While the virtual docking experiments were able to reproduce the binding orientation of moenomycin, a degree of uncertainty remained due to the flexibility of the binding site loop (Fig. 7), and its ability to adopt different conformations depending on the ligand 49. However, the docking experiments clearly indicated the potential of the inhibitors to extend to the acceptor binding site not occupied by moenomycin.

### *In vivo* studies

ACL20215 testing with *in vitro* metabolic stability assays showed no degradation of the compound using both human and mouse liver microsomes. The *in vivo* pharmacokinetic properties of ACL20215 and ACL20964 were investigated using intravenous (i.v.) administration at 3.5 mg kg^−1^ to male rats (Table 2 and Supplementary Table 4). Both compounds had a high apparent half-life (*t*_1/2_) of 27.2 and 33.8 h, respectively. They also showed a very high volume of distribution (*V*_D_) and a high clearance (Cl_total_). No urinary excretion was detected for either compound, and no metabolites were observed in plasma. The maximum tolerated dose for both compounds (see Supplementary methods) was determined following intraperitoneal (i.p.) administration of the compounds to mice, and showed good tolerance up to 60 mg kg^−1^, with no mortality up to 100 mg kg^−1^. Both compounds induced some minor changes to central/autonomic responses at the higher dose of 100 mg kg^−1^. No adverse effects were observed in a single-dose study (4 mg kg^−1^) following i.v. administration.

ACL20215 and ACL20964 were subsequently tested in a mouse model of septicemia, using 10 male CD-1 (*Crl.*)-derived mice, inoculated i.p. with a LD_90–100_ of *S. aureus* (Smith; 3.7 × 10^5^ c.f.u. per mouse). Both compounds administered i.p. 10 min after inoculation at 50 mg kg^−1^ resulted in 100% survival rate after 7 days. However, same studies with i.v. administration of the compounds (4 mg kg^−1^, 10 min after inoculation) showed no antibacterial effect, resulting only in a 10% survival rate, compared with 90% for ampicillin (0.1 mg kg^−1^). The lack of efficacy following i.v. administration is most likely due to a lower dose (4 mg kg^−1^, limited by solubility) combined with the high volume of distribution and serum-binding properties of the compounds, which effectively lowers the free drug concentration at the site of infection. When a higher dose (50 mg kg^−1^ as a suspension) was administered i.p. at the site of infection, the high local concentration of the drug ensures effective clearance of the bacterial infection. Further optimization of compound properties, dose or formulation is required for parenteral administration.

**Figure 8:**
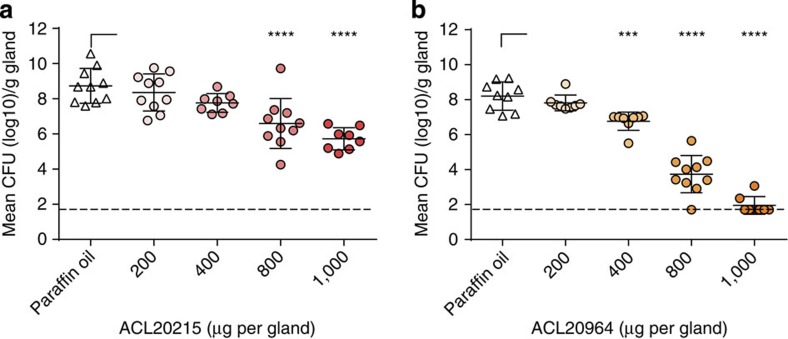
*In vivo* efficacy in mastitis mouse model. *S. aureus* c.f.u. counts (c.f.u. g^−1^ gland) at 14 h post treatment in infected mouse mammary glands treated with increasing doses of ACL20215 (**a**) and ACL20964 (**b**). Significance compared with control (paraffin oil) are given as *P*<0.001 (***) and *P*<0.0001 (****), calculated by one-way analysis of variance followed by Holm–Sidak *post hoc* test. Dashed line represents the detection limit at 1.7 log_10_c.f.u. Data values are given in Supplementary Table 5.

Additional *in vivo* studies were conducted with ACL20215 and ACL20964 using a mouse mammary gland infection (mastitis) model with intraductal inoculation of *S. aureus* (Newbould 305, ATCC 29740). Each compound was instilled at different doses into the teat canal of both contralateral glands from the fourth mammary gland pair of lactating mice at 4 h after bacterial inoculation. Mice were killed at 14 h post infection and both glands were analyzed for c.f.u. counts (Fig. 8, Table 2 and Supplementary Tables 5 and 6). The effective dose to reduce the bacterial load by 2 × log(c.f.u.) (ED_2log_) were 730 and 510 μg per gland, for ACL20215 and ACL20964, respectively, indicating that both compounds cleared 99% of the staphylococci from the infected mammary gland at a moderate dose. Similarly, the protective doses to clear all bacteria in 50% (PD_50_) and 100% (PD_100_) of the glands, respectively, indicated that a high dose of ACL20964 very efficiently cleared *S. aureus* from the infected glands (Table 2). In contrast, no PD values could be determined for ACL20215 as the latter compound was unable to eradicate all bacteria in 50 or 100% of the glands in the analyzed doses to at least the detection limit of the assay.

## Discussion

Using moenomycin A and previously reported GT inhibitors^27^,^28^, we designed and synthesized a small library of disaccharide-based compounds with a smaller, more drug-like, hydrophobic tail^29^. These compounds (such as ACL19378) showed good *in vitro* antibacterial activity but had unfavourable physicochemical properties that limited their *in vivo* application. Nevertheless, this set of active compounds gave valuable structure–activity relationship information, which was used to design libraries of compounds based on a smaller monosaccharide scaffold^37^. This strategy also reduced compound hydrophobicity and chemical complexity, enabling the synthesis of the first reported direct GT inhibitors with *in vivo* efficacy against bacteria.

A reductionist approach, moving from disaccharide mimics of the moenomycin EFG fragment to a smaller monosaccharide scaffold, maintains the key pyranose scaffold and the substitution pattern derived from the disaccharide actives. Chemical chirality inherent in the pyranose scaffold ensures a rigid three-dimensional positioning of substituents that is maintained in the series. Second, the solid-phase synthetic method allowed us to make substantial libraries of chemotypes designed to mimic the disaccharide series. In this way, we identified a series of compounds, corresponding to the amino-benzimidazole chemotype, which showed clear antibacterial activity against a range of drug-resistant Gram-positive bacteria. While the cell-based activity suggests a preference for more hydrophobic substituents, some structural variations are not reflected in their activity, such as the difference in activity between a 2- and 1-naphthyl group and the lack of activity of the corresponding biphenyl compound.

The two most promising compounds from this monosaccharide library, that is, ACL20215 and ACL20964, showed good *in vitro* antibacterial activity against a range of Gram-positive bacteria, including those resistant to common antibiotics, that is, MRSA, GISA and VanA enterococci. PG biosynthesis assay data, taken together, suggests that both compounds trigger an accumulation of lipid II and a decrease of mature PG, as is the case for moenomycin A. Compounds can inhibit the function of GT with IC_50_ values similar to that determined for moenomycin. The virtual docking experiment suggest that the compounds are able to bind in the catalytic site of the GT by occupying part of the donor lipid II-binding site (similar to moenomycin A) as well as part of the acceptor lipid II-binding site (not occupied by moenomycin A).

Both hit compounds can be tolerated in mice up to a dose of 100 mg kg^−1^, while showing good metabolic stability in rats. Even though the library design aimed to reduce the lipophilicity, it is apparent that GT inhibitory compounds require a certain degree of hydrophobicity to be active *in vitro* and *in vivo*. The monosaccharide scaffold is an excellent scaffold for drug design, as it is able to present various substituents or binding elements (in this case, three hydrophobic elements) in diverse spatial orientation using up to five chiral attachment points^38^. The scaffold is also able to present those substituents in a conformational rigid form, indicated by the fact that both monosaccharide compounds, ACL20215 and ACL20964, existed as two atropisomers^55^,^56^ (see Supplementary Fig. 1 and Supplementary Note 1), conformational restricted isomers or rotamers, which would not occur if the carbohydrate scaffold itself was flexible. Virtual docking experiments show both atropisomers among the top ranked poses. It is reasonable to assume that one isomer will be the preferred binding partner for the GT active site, but our *in silico* and *in vitro* experiments were unable to distinguish them.

The membrane-associated nature of the GT enzyme and the hydrophobicity of its natural substrate lipid II necessitates a certain degree of lipophilicity for a compound with an inhibitory effect. While serum binding could not be eliminated in this pilot series, ACL20215 and ACL20964 showed *in vivo* efficacy without toxicity. GT inhibition hence remains a very attractive drug discovery target^3^, as the current inhibitor moenomycin shows extremely low induction of antibiotic resistance^14^,^15^, and also inhibits the conjugative transfer of resistance plasmids^19^,^57^; significant advantages given the current background of increased antimicrobial resistance.

## Methods

### Solid-phase synthesis

All monosaccharide compounds were synthesized on solid-phase using an orthogonally protected galactitol-building block attached to WANG resin. The synthesis of ACL20215 and ACL20964 is given in Supplementary Fig. 9 and below as a representative example.

DTPM removal: the resin was treated with a solution of 5% hydrazine hydrate in dimethylformamide (DMF; 10 ml g^−1^ of resin), shaken (1 h, RT), drained and washed (3 × DMF, 3 × DCM, 3 × DMF). Urea formation: the resin was treated with a solution of 4-chloro-3-trifluoromethyl-phenyl isocyanate (0.15 M) in DMF (10 ml g^−1^ of resin), shaken (O/N, RT), drained and washed (3 × DMF, 3 × DCM). The resin was taken up in a solution of sodium methoxide (0.15 M) in MeOH (5 ml g^−1^ of resin) and tetrahydrofuran (20 ml g^−1^ of resin), shaken (3 h, RT), drained and washed (3 × tetrahydrofuran, 3 × MeOH, 3 × DCM, 3 × DMF). Azide reduction: the resin was treated with a solution of lithium *tert*-butoxide (0.2 M) and DL-dithiothreitol (DTT, 0.2 M) in DMF (15 ml g^−1^ of resin), shaken (O/N, RT), drained and washed (3 × DMF, 3 × MeOH, 3 × DCM, 3 × DMF).

Formation of the substituted benzimidazoles: the resin was treated with a solution of 4-fluoro-3-nitro-benzotrifluoride (0.36 M) and DIPEA (0.36 M) in DMF (10 ml g^−1^ of resin), and heated at 50 °C. The resin was drained and washed with (3 × DMF, 3 × DCM, 3 × DMF). Reduction the nitro group: the resin was treated with a solution of SnCl_2_.2H_2_O in DMF (2.0 M, 10 ml g^−1^ of resin), shaken (O/N, RT), drained and washed (3 × DMF, 3 × DMF/MeOH 1:1, 3 × DCM, 3 × DMF, 3 × DMF/MeOH 1:1, 3 × DCM).

To form the benzimidazole, the resin was treated with DIPEA (0.5 M) in DCM (10 ml g^−1^ of resin), shaken (1 h, RT), drained and washed (3 × DCM), followed by a solution of cyanogen bromide (1.0 M, 10 ml g^−1^ of resin), shaken (O/N, RT), drained and washed (3 × DCM, 3 × MeOH, 3 × DCM). To alkylate the 2-amine, the resin was treated with a solution of benzyl bromide or 1-(bromomethyl) naphthalene (0.4 M) and DIPEA (0.8 M) in DMF (10 ml g^−1^ of resin), shaken (O/N, RT), drained and washed (3 × DCM, 3 × MeOH, 3 × DCM).

Cleavage and purification: each resin was treated with 10% TFA, 20% triethylsilane in dry DCM (1.5 ml), allowed to stand at RT for 3 h, drained into a test tube and washed (3 × DCM). The concentrated samples were treated with a solution of saturated ammonia in methanol (1.0 ml) and left to stand at RT for 2 h, and concentrated by vacuum. Crude samples were purified using preparative high-performance liquid chromatography on a C-18 column (water/acetonitrile gradient).

### Analytical data for ACL20215

The analytical data for ACL20125 are given as ^1^H-NMR, temperature dependent ^1^H-NMR, ^13^C-NMR, COSY, edCOSY and HMBC NMR spectra in Supplementary Figs. 10–15, respectively. The structure of ACL20215 exists as two distinctive rotamers or conformational isomers that can be detected in NMR experiments. Transition between the two isomers, or atropisomers, can be achieved by heating the sample to 45 °C (see Supplementary Fig. 11). *In silico* analysis of the structure and conformation of ACL20125 indicate restricted torsional rotation of the C^4^–N^Benzimidazole^ bond, due to size of the benzimidazole group. Energy barrier calculation indicate an upper range of 25 kcal/mol for this rotational barrier (see Supplementary Fig. 3), which, in relation to other known atropisomers, corresponds to an interconversion rate from a few hours to a few days^56^.

^1^H-NMR (600 MHz, dimethylsulphoxide (DMSO)-d6): major rotamer *δ* 9.20 (br s, 1H, 7-NH), 8.10 (d, *J*=8.0 Hz, 1H, H-20), 8.02 (d, *J*=2.2 Hz, 1H, H-9), 7.50 (d, *J*=8.9 Hz, 1H, H-13), 7.48 (dd, *J*=2.2, 8.9 Hz, 1H, H-12), 7.42 (s, 1H, H-17), 7.39 (d, *J*=7.3 Hz, 2H, H-25, H-29), 7.27 (dd, *J*=7.3, 7.3 Hz, 2H, H-26, H-28), 7.22 (d, *J*=8.0 Hz, 1H, H-19), 7.19 (dd, *J*=7.3, 7.3 Hz, 1H, H-27), 7.14 (t, *J*=5.8 Hz, 1H, 15-NH), 6.34 (d, *J*=6.0 Hz, 1H, 2-NH), 5.41 (d, *J*=5.8 Hz, 1H, 3-OH), 4.91 (m, 1H, H-4), 4.91 (t, *J*=5.1 Hz, 1H, 6-OH), 4.63 (m, 2H, H-23), 4.27 (dd, *J*=5.2, 11.2 Hz, 1H, H-1β), 4.00 (m, 2H, H-3, H-5), 3.87 (dddd, *J*=5.2, 5.8, 11.2, 11.2 Hz, 1H, H-2), 3.40 (m, 1H, H-6a), 3.39 (m, 1H, H-1α), 3.18 (dd, *J*=5.8, 5.8, 11.2 Hz, 1H, H-6b); minor rotamer *δ* 9.25 (br s, 1H, 7-NH), 8.10 (d, *J*=2.2 Hz, 1H, H-9), 7.78 (t, *J*=5.2 Hz, 1H, 15-NH), 7.54 (m, 2H, H-12, H-13), 7.54 (d, *J*=7.3 Hz, 2H, H-25, H-29), 7.45 (s, 1H, H-17), 7.36 (d, *J*=8.0 Hz, 1H, H-20), 7.35 (dd, *J*=7.3, 7.3 Hz, 2H, H-26, H-28), 7.27 (dd, *J*=7.3, 7.3 Hz, 1H, H-27), 7.21 (d, *J*=8.0 Hz, 1H, H-19), 6.36 (d, *J*=6.0 Hz, 1H, 2-NH), 5.29 (d, *J*=6.1 Hz, 1H, 3-OH), 5.00 (m, 1H, H-4), 4.96 (t, *J*=5.2 Hz, 1H, 6-OH), 4.65 (m, 1H, H-23a), 4.63 (m, 1H, H-23b), 4.24 (dddd, *J*=5.2, 5.8, 11.2, 11.2 Hz, 1H, H-2), 4.16 (dd, *J*=5.2, 11.2 Hz, 1H, H-1β), 4.07 (m, 2H, H-3, H-5), 3.48 (ddd, *J*=5.2, 5.2, 11.2 Hz, 1H, H-6a), 3.39 (m, 1H, H-1α), 3.27 (dd, *J*=5.8, 5.8, 11.2 Hz, 1H, H-6b); ^13^C-NMR (150 MHz, DMSO-d6): major rotamer δ 158.9 (C-15), 154.9 (C-7), 143.0 (C-16), 140.1 (C-24), 139.9 (C-8), 137.4 (C-21), 131.8 (C-12), 128.0 (C-26, C-28), 126.8 (C-25, C-29), 126.4 (C-27), 125.5 (q, 1JCF=271 Hz, C-22), 122.9 (q, 1JCF=273 Hz, C-14), 122.6 (q, 2JCF=31 Hz, C-10), 122.3 (C-13), 121.5 (C-11), 121.0 (q, 2JCF=31 Hz, C-18), 116.1 (C-9), 115.0 (C-19), 112.1 (C-20), 111.4 (C-17), 77.7 (C-5), 70.7 (C-3), 68.8 (C-1), 60.3 (C-6), 55.2 (C-4), 48.8 (C-2), 46.0 (C-23); minor rotamer *δ* 155.8 (C-15), 155.0 (C-7), 142.1 (C-16), 140.9 (C-21), 139.9 (C-8), 139.0 (C-24), 131.9 (C-12), 128.4 (C-26, C-28), 127.5 (C-25, C-29), 127.0 (C-27), 125.5 (q, 1JCF=271 Hz, C-22), 122.9 (q, 1JCF=273 Hz, C-14), 122.6 (q, 2JCF=31 Hz, C-10), 122.3 (C-13), 121.5 (C-11), 121.0 (q, 2JCF=31 Hz, C-18), 116.1 (C-9), 115.0 (C-19), 109.4 (C-20), 111.1 (C-17), 77.9 (C-5), 70.8 (C-3), 69.0 (C-1), 59.9 (C-6), 56.4 (C-4), 48.5 (C-2), 46.8 (C-23); HRESIMS (*m*/*z*): [M+H]+ calcd. for C_29_H_27_Cl_1_F_6_N_5_O_4_, 658.1650; found, 658.1659.

### Analytical data for ACL20964

The analytical data for ACL20964 are given as ^1^H-NMR, ^13^C-NMR, COSY, edCOSY and HMBC NMR spectra in Supplementary Figs 16–20, respectively. Similar to ACL20215, ACL20964 exists as two conformational isomer, due to rotational restriction of the C^4^–N^Benzimidazole^ bond, caused by the large benzimidazole group.

^1^H-NMR (600 MHz, DMSO-d6): major rotamer *δ* 9.26 (br s, 1H, 7-NH), 8.14 (d, *J*=8.2 Hz, 1H, H-32), 8.13 (d, *J*=8.2 Hz, 1H, H-20), 8.04 (s, 1H, H-9), 7.94 (d, *J*=8.2 Hz, 1H, H-29), 7.81 (d, *J*=8.2 Hz, 1H, H-27), 7.63 (d, *J*=8.2 Hz, 1H, C-25), 7.54 (m, 2H, H-30, H-31), 7.51 (m, 2H, H-12, H-13), 7.42 (s, 1H, H-17), 7.39 (dd, *J*=8.2, 8.2 Hz, 1H, H-26), 7.22 (d, *J*=8.2 Hz, 1H, H-19), 7.19 (t, *J*=5.7 Hz, 1H, 15-NH), 6.34 (br s, 1H, 2-NH), 5.47 (br s, 1H, 3-OH), 5.09 (m, 2H, H-23), 4.97 (m, 1H, H-4), 4.95 (br s, 1H, 6-OH), 4.28 (dd, *J*=4.8, 11.2 Hz, 1H, H-1β), 4.01 (m, 2H, H-3, H-5), 3.92 (m, 1H, H-2), 3.45 (m, 1H, H-6a), 3.39 (m, 1H, H-1α), 3.23 (m, 1H, H-6b); minor rotamer *δ* 9.26 (br s, 1H, 7-NH), 8.21 (d, *J*=8.2 Hz, 1H, H-32), 8.10 (s, 1H, H-9), 7.96 (d, *J*=8.2 Hz, 1H, H-29), 7.87 (d, *J*=8.2 Hz, 1H, H-27), 7.78 (t, *J*=5.1 Hz, 1H, 15-NH), 7.73 (d, *J*=8.2 Hz, 1H, C-25), 7.60 (dd, *J*=8.2, 8.2 Hz, H-31), 7.55 (m, 2H, H-12, H-13), 7.54 (m, 1H, H-30), 7.47 (s, 1H, H-17), 7.50 (m, 1H, H-26), 7.37 (d, *J*=8.2 Hz, 1H, H-20), 7.22 (d, *J*=8.2 Hz, 1H, H-19), 6.36 (br s, 1H, 2-NH), 5.31 (br s, 1H, 3-OH), 5.16 (dd, *J*=5.6, 15.3 Hz, 1H, H-23a), 5.11 (dd, *J*=4.8, 15.3 Hz, 1H, H-23b), 5.01 (m, 1H, H-4), 4.95 (br s, 1H, 6-OH), 4.24 (m, 1H, H-2), 4.16 (dd, *J*=5.2, 11.2 Hz, 1H, H-1β), 4.05 (m, 1H, H-3), 4.01 (m, 1H, H-5),3.48 (m, 1H, H-6a), 3.39 (m, 1H, H-1α), 3.27 (m, 1H, H-6b); ^13^C-NMR (150 MHz, DMSO-d6): major rotamer *δ* 158.7 (C-15), 154.9 (C-7), 143.1 (C-16), 140.0 (C-8), 137.5 (C-21), 134.9 (C-33), 134.2 (C-28), 131.9 (C-12), 130.9 (C-24), 128.5 (C-29), 127.0 (C-27), 126.6 (q, 2JCF=31 Hz, C-10), 126.1 (C-31), 125.6 (C-30), 125.5 (C-26), 125.4 (q, 1JCF=271 Hz, C-22), 124.6 (C-25), 123.4 (C-32), 122.8 (q, 1JCF=273 Hz, C-14), 122.3 (C-13), 121.5 (C-11), 121.0 (q, 2JCF=31 Hz, C-18), 116.1 (C-9), 115.0 (C-19), 112.1 (C-20), 111.4 (C-17), 77.6 (C-5), 70.7 (C-3), 68.8 (C-1), 60.3 (C-6), 55.1 (C-4), 49.0 (C-2), 44.3 (C-23); minor rotamer *δ* 155.8 (C-15), 154.9 (C-7), 142.1 (C-16), 140.9 (C-21), 140.0 (C-8), 134.2 (C-33), 133.2 (C-28), 131.8 (C-12), 130.9 (C-24), 128.6 (C-29), 127.7 (C-27), 126.6 (q, 2JCF=31 Hz, C-10), 126.5 (C-31), 125.8 (C-30), 125.6 (C-26), 125.4 (q, 1JCF=271 Hz, C-22), 125.4 (C-25), 123.4 (C-32), 122.8 (q, 1JCF=273 Hz, C-14), 122.3 (C-13), 121.5 (C-11), 121.0 (q, 2JCF=31 Hz, C-18), 116.1 (C-9), 115.0 (C-19), 111.1 (C-17), 109.5 (C-20), 77.8 (C-5), 70.9 (C-3), 68.5 (C-1), 59.8 (C-6), 56.4 (C-4), 48.5 (C-2), 44.7 (C-23); HRESIMS (*m*/*z*): [M+H]+ calcd. for C_33_H_29_Cl_1_F_6_N_5_O_4_, 708.1807; found, 708.1797.

### Virtual docking

The virtual docking of inhibitor and moenomycin structures into the binding site of MGT from *S. aureus* were done using the software Glide (version 6.5, Schrödinger, LLC, New York, 2014 (ref. 58)) and using several different crystal structures with different binding site loop conformations. In addition, the induced fit protocol in the Schrödinger software package (Induced Fit Docking protocol 2014-4, Glide version 6.5, Prime version 3.7, Schrödinger, LLC^59^,^60^) was used to allow conformational flexibility of the GT domain. The docking experiments were all done using the standard precision (SP) in Glide and defining a binding site with 13 Å around the crystal structure of moenomycin, large enough to include also the acceptor site which is not occupied by moenomycin. For the structure of moenomycin, a truncated version was used, without the fatty acid chain and only a lactic acid attached to the phosphate group. Models for inhibitors were built in four different conformations, ^4^C_1_ and _1_C^4^ chair conformation for the galactose moiety, and two different orientations for the benzimidazole moiety compared with the galactose ring. All structure models were built using Maestro (version 10.0, Schrödinger, LLC, New York, 2014).

The different approaches, that is, crystal structures and protocols, were validated and the best one selected by comparing the docking orientation of moenomycin with the one found in the crystal structures, by visual inspection. The following models for MGT from *S aureus* have been used for the docking experiments: 3HZSm–3HZS^48^ changing to wild type, by mutating Asn100 to Asp, 3VMRm–3VMR^49^ modelling the missing loop residues (2 residues) with loop search, 3NB6sa-Homology model of MGT *S. aureus* sequence using 3NB6 (ref. 11) (PGT from *A. aeolicus*) with complete loop as template, 3VMSm–3VMS^49^ modelling missing loop (7 residues) using the loop in 3NB6 (ref. 11) as template.

From these receptor models, 3HZSm was able to reproduce the binding orientation of moenomycin A as found in the crystal structure (see Supplementary Fig. 25; a, green: crystal structure; grey; docked structure). The 3VMRm model produced similar orientations for moenomycin but with different orientation of the D ring. In 3NB6sa model, moenomycin was binding in the same binding pocket but its orientation was always different, with phosphoglycerate (G) portion of the molecule facing either to the solvent, the donor or acceptor binding site. In the 3VMSm model, which has the most open groove between the donor and acceptor site, moenomycin was actually oriented partly across the acceptor binding site. Induced fit protocol on those models did not produce better moenomycin binding orientations compared with the standard docking protocol.

### *In vitro* PG biosynthesis

The cell-free particulate fraction of *B. megaterium* KM (ATCC13632), capable of catalysing the polymerization of PG from UPD-linked precursors was performed as described previously^61^. *B. megaterium* was grown in standard medium, harvested and washed with Tris-buffer by centrifugation. Resuspended bacteria were subjected to three freeze/thaw cycles (5 min dry ice, followed by 10 min at RT), homogenized by the glass homogenizer and centrifuged all at 4 °C, leaving most of the cell wall in the pellet. Resuspended pellet was combined with UDP-*N*-acetylmuramyl-pentapeptide, [^14^C]UDP-*N*-acetylglucosamine and individual compounds or antibiotics (that is, vancomycin hydrochloride or moenomycin A), and incubated at for 3 h at RT, placed in a boiling water bath for 3 min to inactivate enzymes and to prevent any further lipid II transformation, and analysed by TLC on silica gel plates. After separation, plates were dried, exposed to phosphorimaging screen (1 week), scanned by Typhoon 8600 calculating the integrated density value of each band on silica gel. Changes of PG or lipid II were calculated as a percentage from negative control (for more details see Supplementary Methods).

### Inhibition of glycosyltransferase

Inhibition of glycosyltransferase was measured using a fluorescence detection method^62^ by adding 1 μM *S. aureus* MGT to 1.45 μM fluorescent dansyl-Lys Lipid II and different concentration of inhibitors, all in a buffer of 50 mM Tris pH 8 containing 10 mM MnCl_2_, 0.08% (v/v) decyl PEG, 10% (v/v) DMSO, 100 μg ml^−1^ hen egg-white lysozyme, in 96-well microtiter plates. Initial rates were measured as a decrease in fluorescence (ex/em: 340/521 nm) and calculated as a percentage compared with no inhibitor as negative control (0% of rate inhibition) and moenomycin A (Sigma, Cat. no.: 32404) as positive control (100% of rate inhibition). The data were fitted to a simple saturation model of inhibitor binding to a single site, from which IC_50_ values were extracted. Moenomycin A showed an IC_50_ of 5 μM in this assay.

### Minimal inhibitory concentration (MIC) determination

The compounds along with standard antibiotics were serially diluted twofold across the wells of 96-well standard polystyrene non-treated plates (Corning 3370). Compounds and standard antibiotic controls ranged from 1.28 mg ml^−1^ to 0.06 μg ml^−1^ with final volumes of 50 μl per well. Bacteria were cultured in Brain–Heart Infusion (Bacto laboratories, Cat. no. CM1135B) at 37 °C overnight. A sample of each culture was then diluted 40-fold in fresh brain–heart infusion broth and incubated at 37 °C for 2–3 h. The resultant mid-log phase cultures were diluted to 5 × 10^5^ c.f.u. ml^−1^ then 50 μl was added to each well of the compound-containing 96-well plates giving a final compound concentration range of 64 μg ml^−1^ to 0.03 μg ml^−1^ in 2.5 × 10^5^ c.f.u. ml^−1^. All the plates were covered and incubated at 37 °C for 24 h. MICs were determined visually as the lowest concentration showing no visible growth.

Antibiotic control compound vancomycin (Sigma, Cat. no.: 861987) was prepared as water solution, while moenomycin A (Sigma, Cat. no.: 32404) was dissolved in DMSO and 20 mM ammonium acetate, due to solubility issues. The average MIC for moenomycin for *S. aureus* was, however, within the range of recent literature^35^.

### *In vivo* mouse mammary gland infection model

For the infection of the mice, *S. aureus* Newbould 305 (ATCC 29740) isolated from a clinical mastitis cases^63^ was used and prepared. The procedure for mouse mammary gland infection has been recently described^64^. CD-1 lactating mice were utilized 12–14 days after giving birth, with pups weaned 1–2 h before bacterial inoculation of the mammary glands. Inoculation of both left (L4) and right (R4) glands of the fourth abdominal mammary gland pair of anesthetized mice with 150 c.f.u. of *S. aureus*, was done using 32-gauge syringes (blunt needle). The antimicrobial formulation was instilled into the mammary gland of anesthetized mice at 4 h after bacterial inoculation, followed by i.p. administration of postoperative analgesic Buprecare. Mice were killed 14 h post treatment, mammary glands (two per mouse) were harvested, weighed and homogenized. Bacterial c.f.u. counts were obtained after quantification of serial logarithmic dilutions of mammary gland homogenates on TSA. The detection limit (DL) was 1.7 log_10_ c.f.u. g^−1^ gland weight (for more details see Supplementary Methods). The animal experiments were approved by the Ethical Committee of the Faculty of Veterinary Medicine, Ghent University (EC2009/133).

## Additional information

**How to cite this article**: Zuegg, J. *et al.* Carbohydrate scaffolds as glycosyltransferase inhibitors with *in vivo* antibacterial activity. *Nat. Commun.* 6:7719 doi: 10.1038/ncomms8719 (2015).

## Supplementary Material

Supplementary Information Supplementary Figures 1-26, Supplementary Tables 1-6, Supplementary Note 1, Supplementary Methods and Supplementary References

## Figures and Tables

**Table 1:** *In vitro* activity data of ACL20215 and ACL20964.

**Organism**		**Strain/type**	**Vancomycin**	**Moenomycin A**	**ACL20215**	**ACL20964**
*MIC* *(μg ml^−1^)*						
*S. aureus*		MSSA, ATCC 25923	1		4	4
		MRSA, ATCC 43300	1	4	4	8
		Newbould 305			2	1
		NRS 17—GISA	8	16–32	8	32
		NRS 1—GISA	4	1	4	16
		VRS 1	>64	8	4	8
		mMRSA, DRSA, *ci*	4	16	4	8
* E. faecium*		ATCC 35667			16	2
		VanA, ATCC 51559	>64	32	8	>64
* E. faecalis*		ATCC 29212			4	8
		VanA, *ci*	>64	>64	8	64
* S. pneumoniae*		MDR, ATCC 700677	2	8–16	4	8–16
* E. coli*		ATCC 25922	>64	>64	>64	>64
*Mutation frequency*						
* S. aureus (at 4 × MIC)*		ATCC 13709			2.5 × 10^−10^	
*HC*_*50*_ *(μg ml^−1^)*						
*Human*		RBC			74	>100
*IC*_*50*_ *(μg ml^−1^)*						
*S. aureus*		MGT			17.1	11.1

*ci*, clinical isolate; DRSA, daptomycin-resistant *S. aureus*; GISA, glycopeptide-intermediate *S. aureus*; HC_50_, half maximal haemolytic concentration; IC_50_, half maximal inhibitory concentration; MDR, multi-drug-resistant; MGT, monofunctional glycosyltranferease; MIC, minimum inhibitory concentration; mMRSA, multi-drug-resistant methicillin-resistant *S. aureus*; Moenomycin A (Sigma, 32404); MRSA, methicillin-resistant *S. aureus;* MSSA, methicillin-sensitive *S. aureus;* RBC, red blood cells; Vancomycin (Sigma, 861987); VRS, vancomycin-resistant *S. aureus*.

All values are μg ml^−1^.

**Table 2:** Pharmacokinetic properties and *in vivo* efficacy of ACL20215 and ACL20964.

	**ACL20215**	**ACL20964**
*Pharmacokinetic properties*		
Metabolic stability (*in vitro*)	No degradation	ND
Rat (i.v.)		
Dose (mg kg^−1^)	3.5	3.5
*t*_1/2_ (h)	27.2	33.8
Plasma Cl_total_ (ml min^−1^ kg^−1^)	42.1	17.9
Blood Cl_total_ (ml min^−1^ kg^−1^)	48.9	21.5
*V*_Z_ (l kg^−1^)	97.2	53.0
Mice (i.p.)		
MTD (mg kg^−1^)	100	100
		
In vivo *efficacy*		
Mouse (i.p.)		
Survival 7 days (%)	100	100
Mouse (i.v.)		
Survival 7 days (%)	10	10
Mouse (mastitis)		
ED_2logc.f.u._ (μg per gland)	730	510
ED_4logc.f.u._ (μg per gland)	1,400	770
PD_50_ (μg per gland)	>1,000	800–1,000
PD_100_ (μg per gland)	>1,000	>1,000

Cl, clearance; ED_2logc.f.u._, effective dose to reduce bacterial load by 2 × log(c.f.u.); ED_4logc.f.u._, effective dose to reduce bacterial load by 4 × log(c.f.u.); i.p., intraperitoneal injection; i.v., intravenous injection; MTD, maximal tolerated dose; ND, not determined; PD_50_, 50% protective dose; PD_100_, 100% protective dose; *t*_1/2_, half-life; V_Z_, volume of distribution.
