# Supplementary material for: Carbohydrate scaffolds as glycosyltransferase inhibitors with in vivo antibacterial activity
Source: Nat Commun. 2015 Jul 21;6:7719. doi: 10.1038/ncomms8719 (PMC4530474; doi:10.1038/ncomms8719)
Supplement: Supplementary Information — Supplementary Figures 1-26, Supplementary Tables 1-6, Supplementary Note 1, Supplementary Methods and Supplementary References [file ncomms8719-s1.pdf]

**Supplementary Figures**

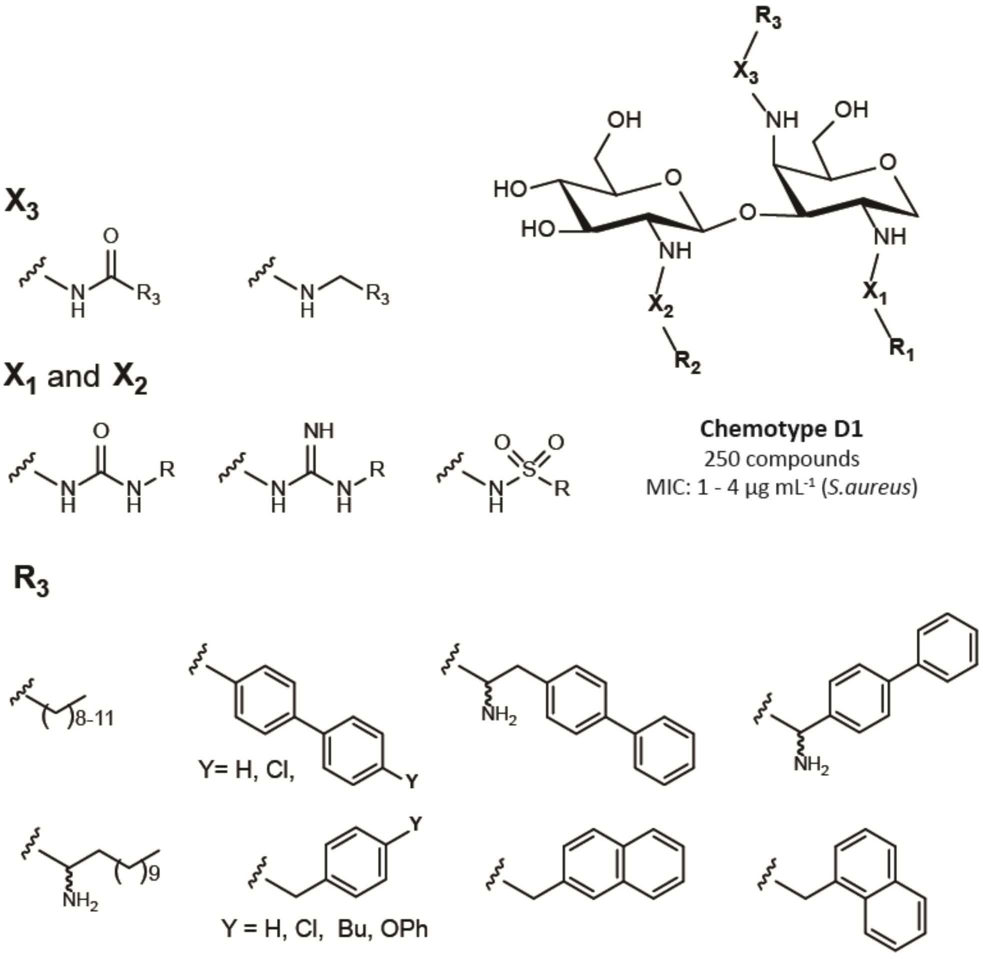


**Supplementary Figure 1**: Design of disaccharides library (Chemotype D1).

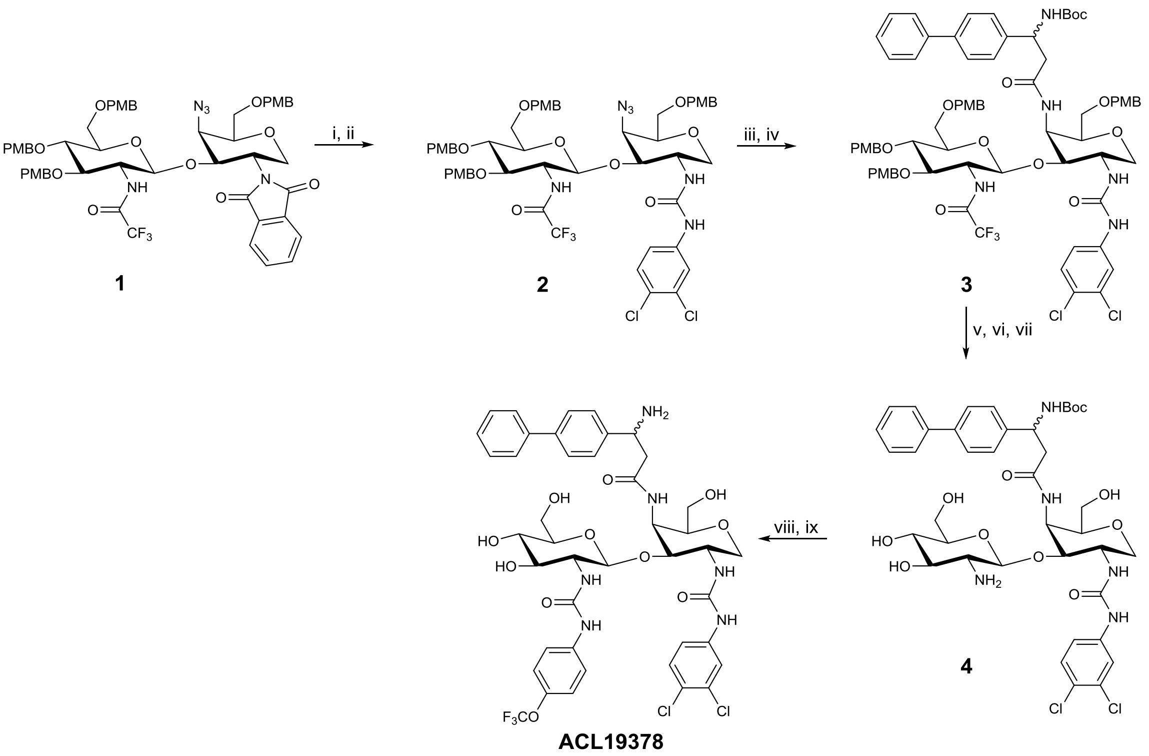


**Supplementary Figure 2:** Synthesis route of disaccharide compound ACL19378.

i) Ethylene diamine, *n*-butanol, 120 °C, 40 min. ii) 3,4-dichlorophenyl isocyanate (1.2 eq) in DMF, 3 h iii) Zn, $NH_4Cl$, H2O, MeOH, o/n iv) 3-([1,1'-biphenyl]-4-yl)-3-((*tert*-butoxycarbonyl)amino)propanoic acid (1.25 eq), HBTU (1.25 eq), DIPEA (4.2 eq), DMF, o/n v) 5% TFA, 15% TES in DCM, 5 h vi) $Boc_2O$ (2 eq), $H_2O$, $NaHCO_3$ (pH 7-8), o/n vii) sat. $NH_3$, MeOH, 55 °C, 5 h viii) 4-(trifluoromethoxy)phenyl isocyanate (1.2 eq) in DMF, o/n ix) 5% TFA, 15% TES in DCM, 2 h ×) sat. $NH_3$, MeOH.

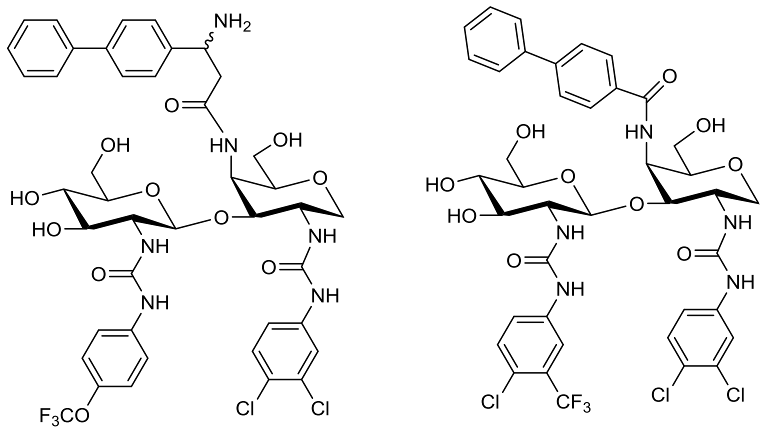

**ACL19378**
MIC: 2 µg $mL^{-1}$ (*S. aureus*)

**ACL19333**
MIC: 2 µg $mL^{-1}$ (*S. aureus*)

**Supplementary Figure 3**: Representative compounds from disaccharide library (Chemotype D1).

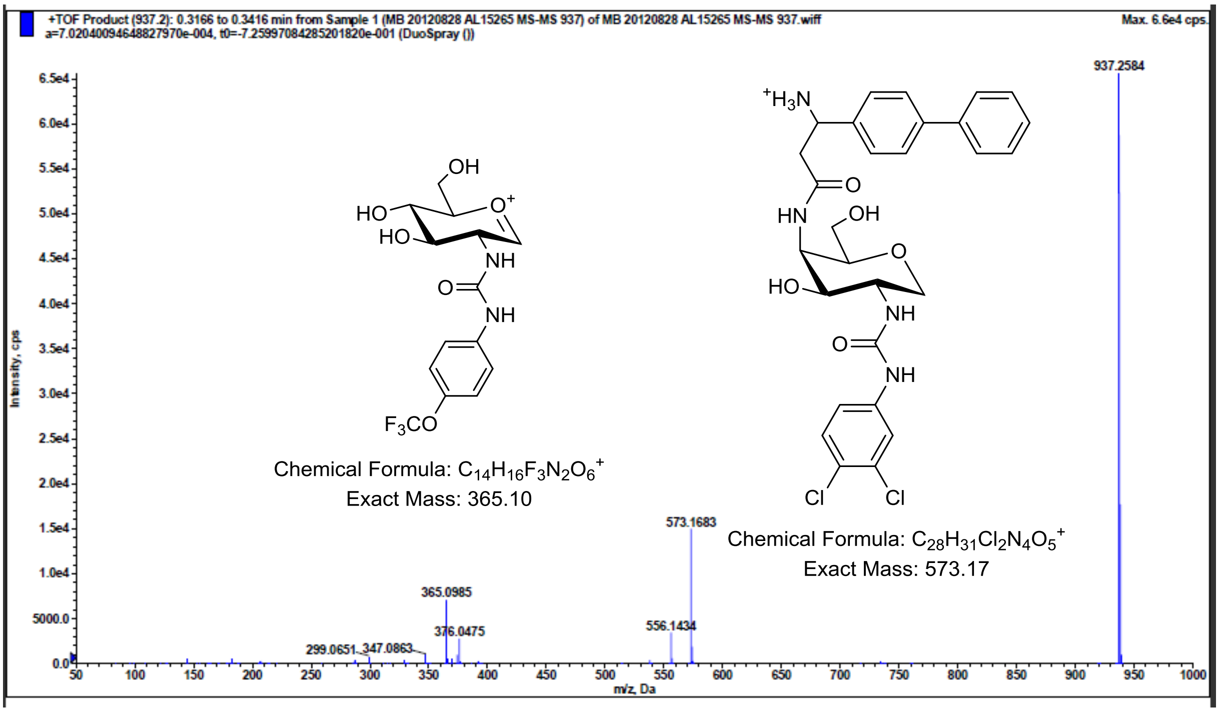


**Supplementary Figure 4:** (+)-ESI-QToF MS/MS data of ACL19378
The spectra is showing the fragmentation into the two saccharide units, shown with the structure

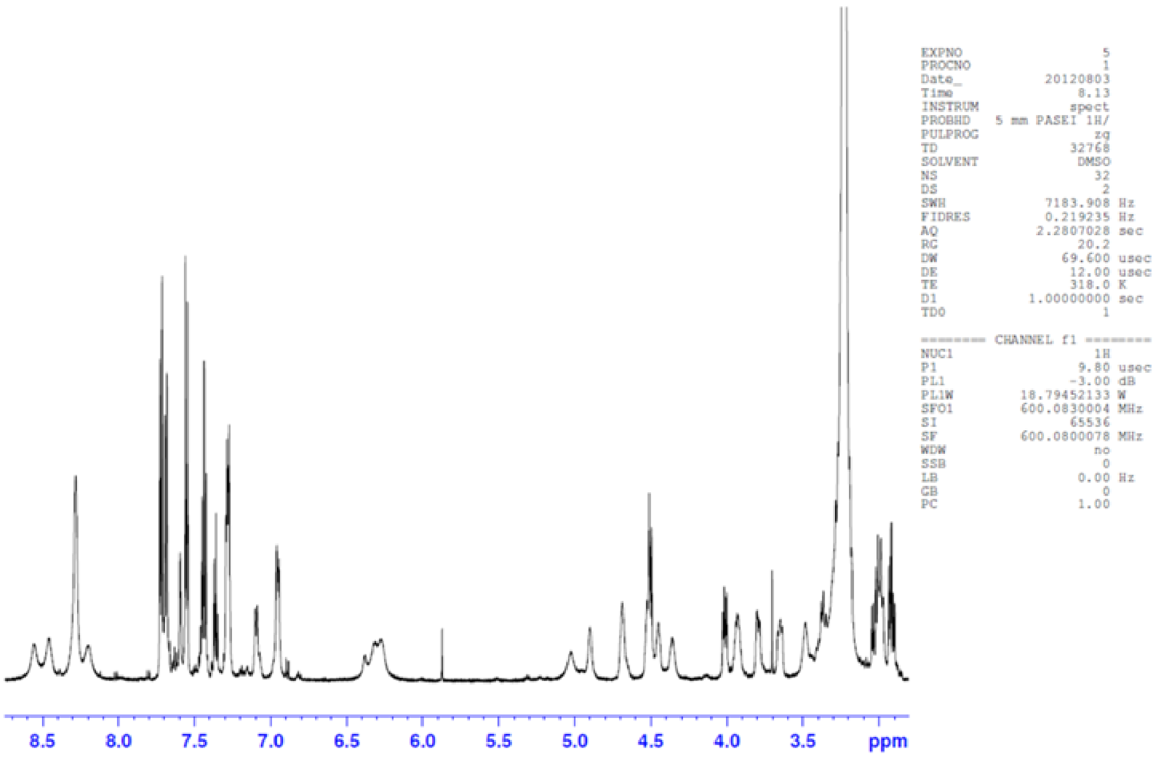


**Supplementary Figure 5**: $^{1}$H NMR spectrum of ACL19378 in DMSO-$d_6$ recorded at 318K.

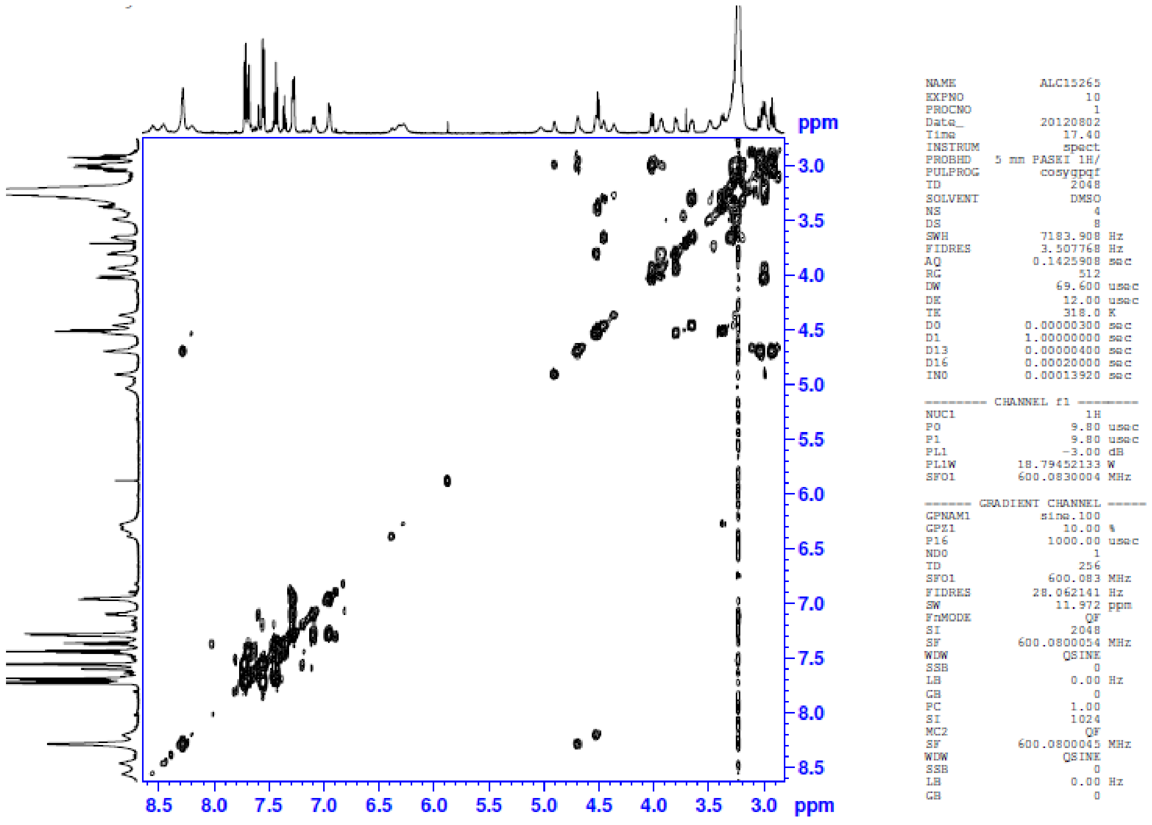


**Supplementary Figure 6**: COSY NMR spectrum of ACL19378 in DMSO-$d_6$ recorded at 318K.

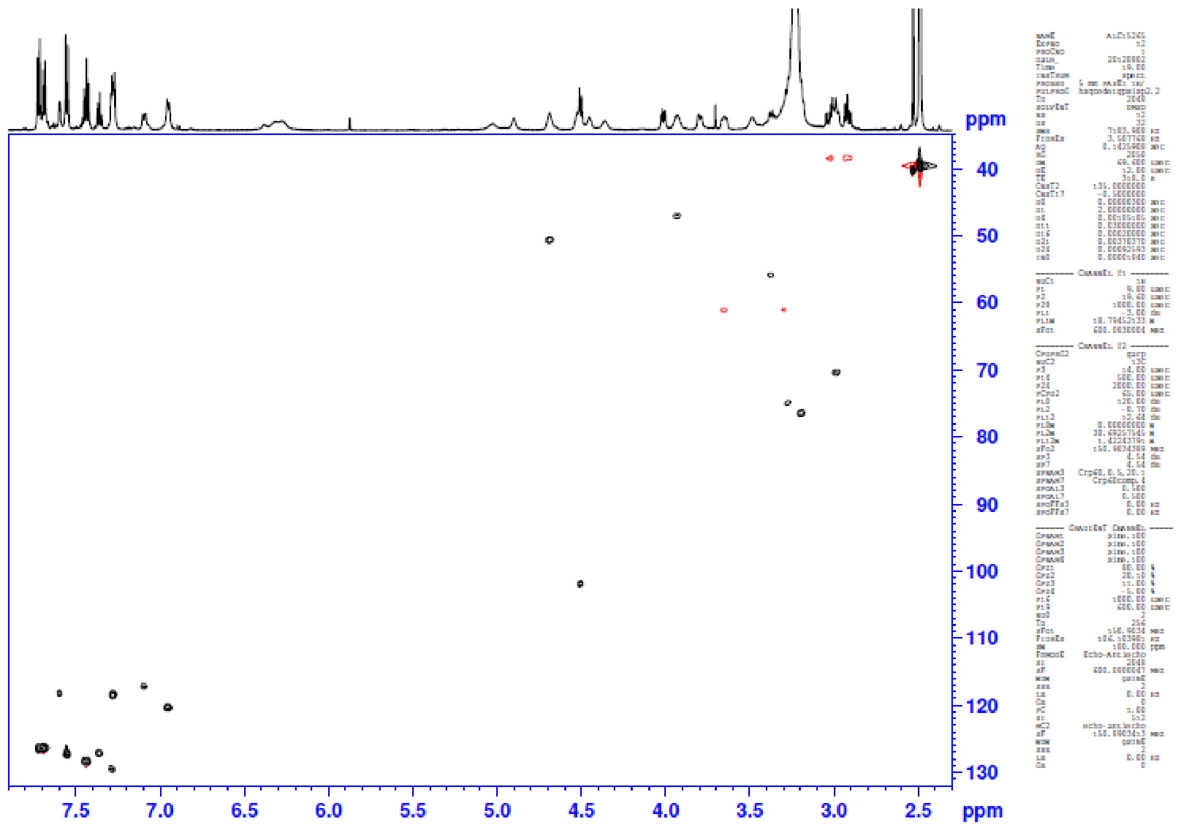


**Supplementary Figure 7**: edHSQC NMR spectrum of ACL19378 in DMSO-$d_6$ recorded at 318K.

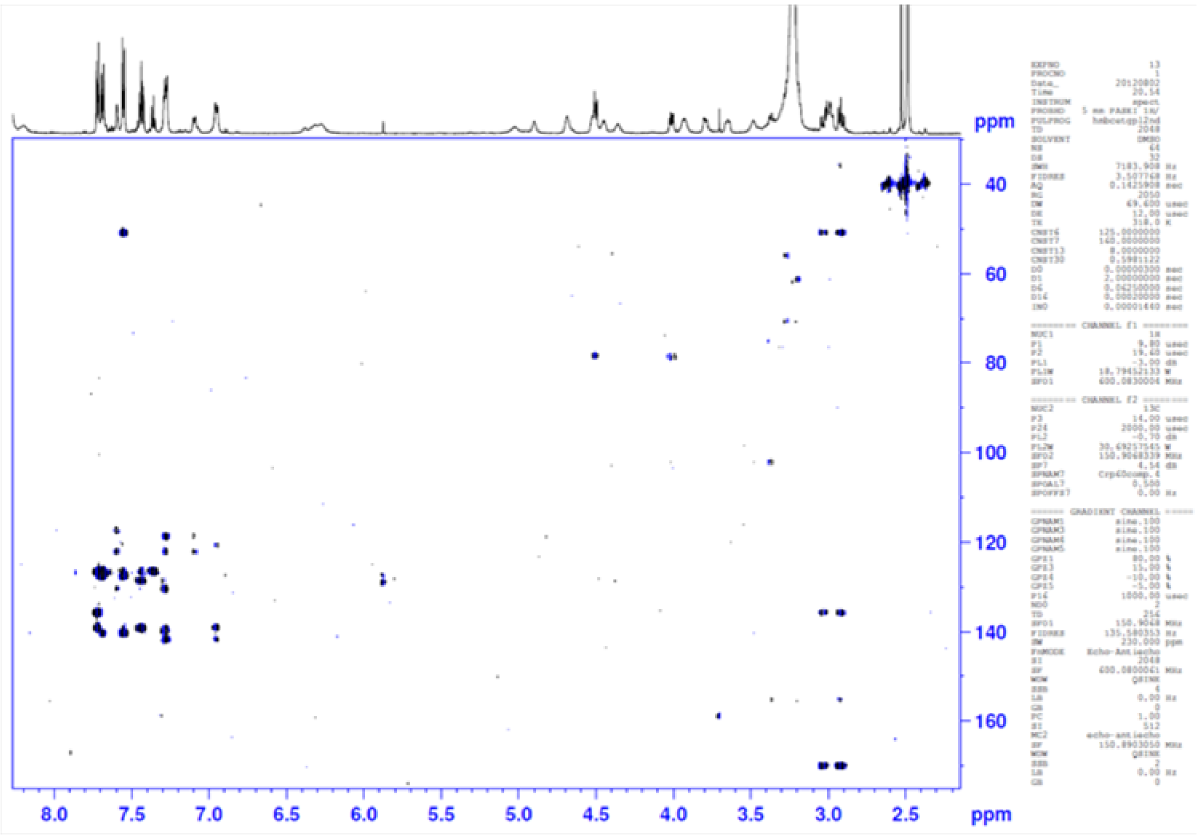


**Supplementary Figure 8**: HMBC NMR spectrum of ACL19378 in DMSO-$d_6$ recorded at 318K.

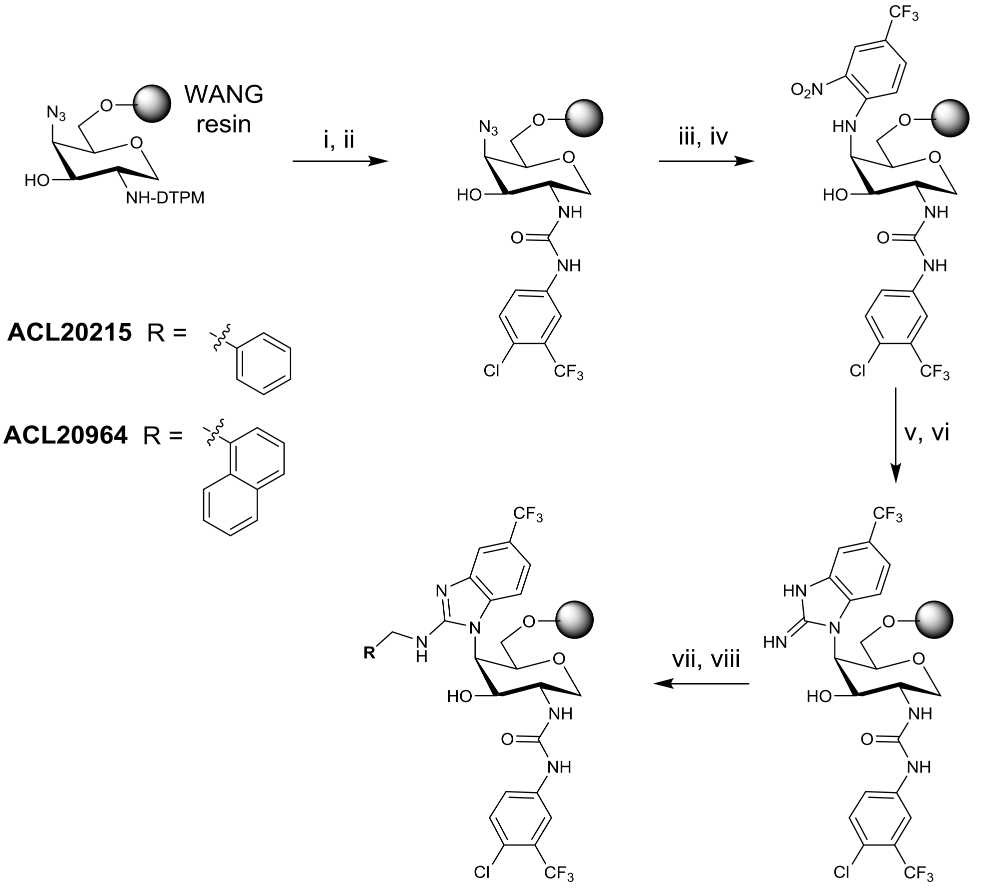


**Supplementary Figure 9**: **Synthesis route for ACL20215 and ACL20964.**
i) 5% $H_2NNH_2.H_2O$ in DMF, 1.5 h ii) a) 4-chloro-3-(trifluoromethyl)phenyl isocyanate in DMF, o/n b) NaOMe (0.15 M) in MeOH/THF (1:4) iii) DTT (0.2 M), $LiO^tBu$ (0.2 M) in DMF, o/n iv) 4-fluoro-3-nitrobenzotrifluoride (5 eq), DIPEA (0.25 M) in DMF, 50 $^oC$, o/n v) $SnCl_2.2H_2O$ (2.0 M) in DMF, o/n vi) a) DIPEA (0.5 M) in DCM, 1 h b) CNBr (1.0 M) in DCM, o/n vii) $RCH_2Br$ (5 eq, 0.4 M), DIPEA (10 eq, 0.8 M) in DMF, o/n viii) a) 10% TFA, 20% TES in DCM, 3 h b) sat. $NH_3$ in MeOH, 2 h.

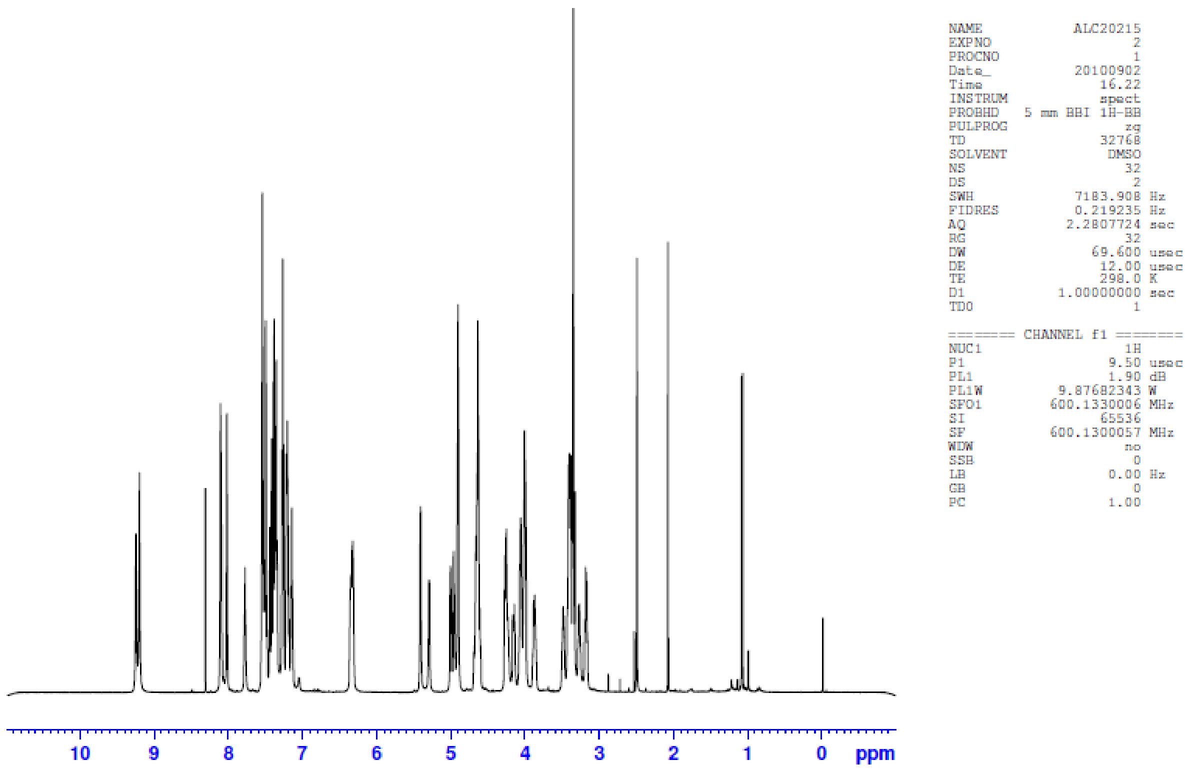


**Supplementary Figure 10**: $^{1}H$ NMR spectrum of ACL20215 in DMSO-$d_6$ recorded at 298K.

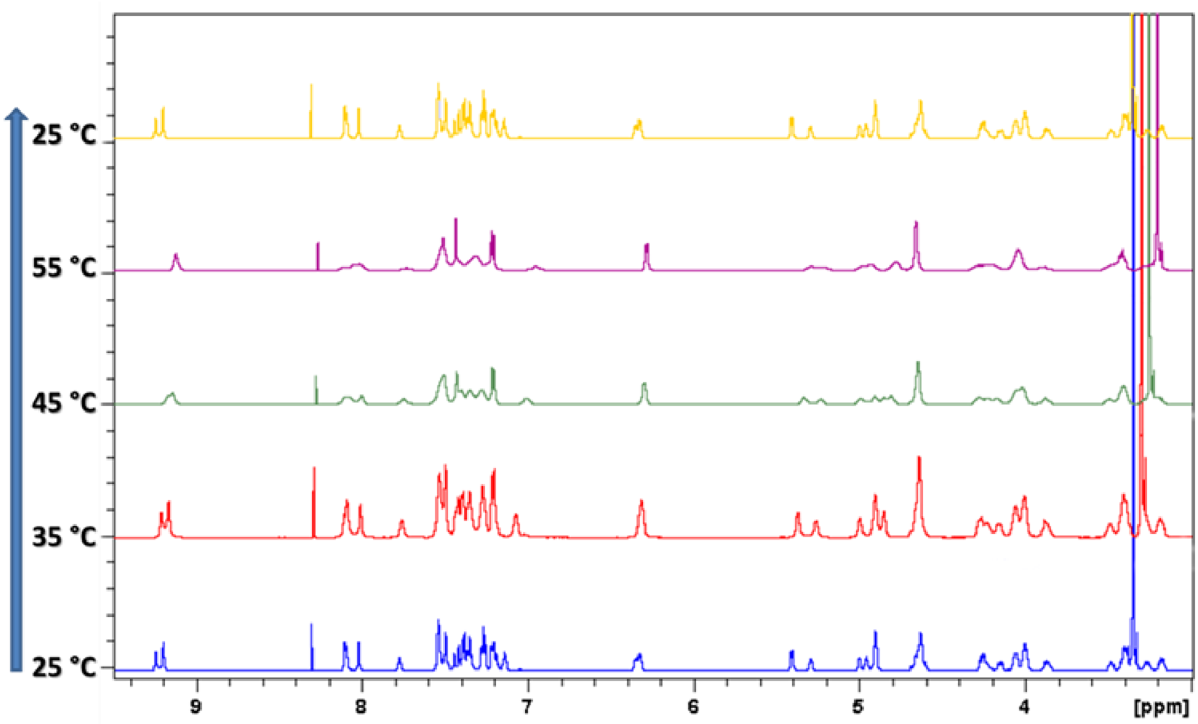


**Supplementary Figure 11**: $^{1}H$ NMR spectra of ACL20215 recorded at different temperatures.

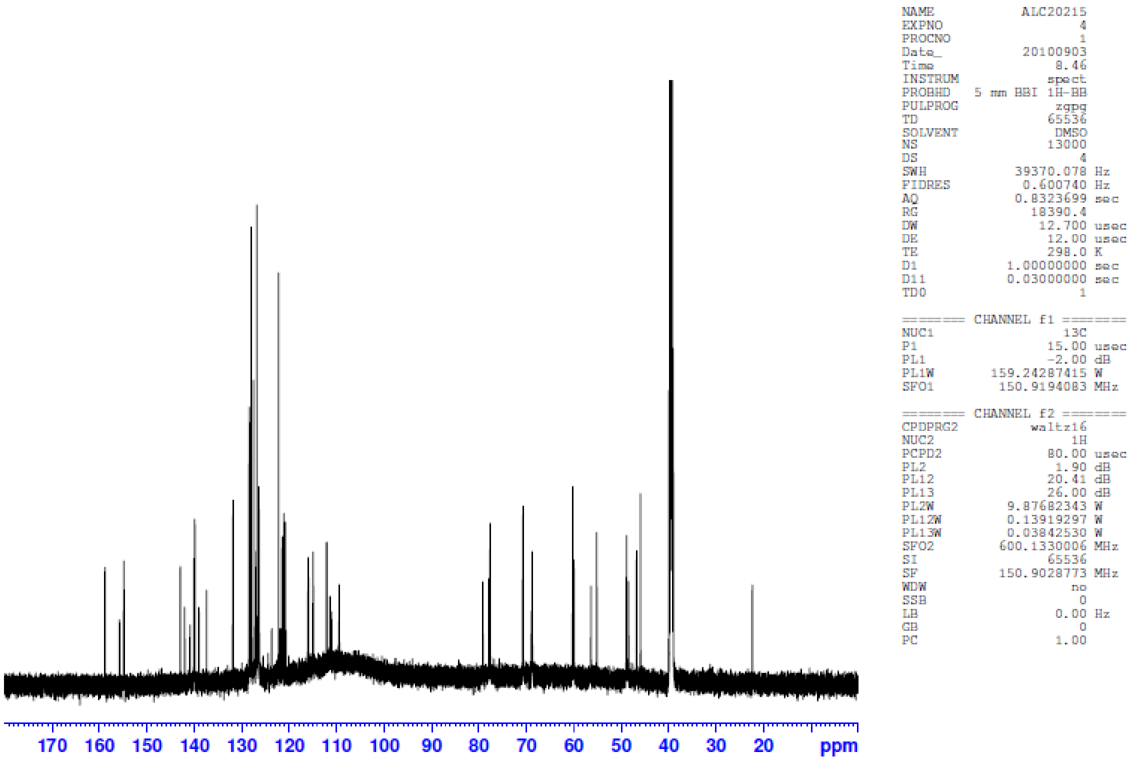


**Supplementary Figure 12**: $^{13}$C NMR spectrum of ACL20215 in DMSO-$d_6$ recorded at 298K.

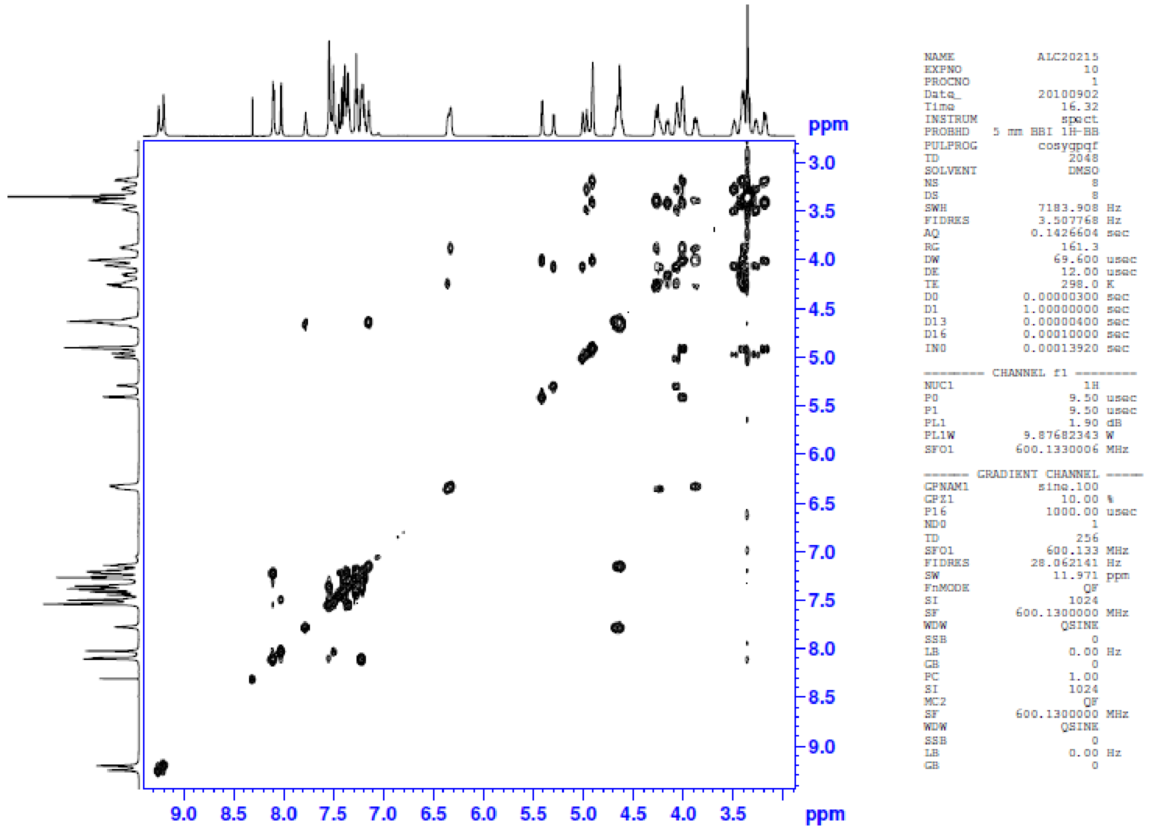


**Supplementary Figure 13**: COSY NMR spectrum of ACL20215 in DMSO-$d_6$ recorded at 298K.

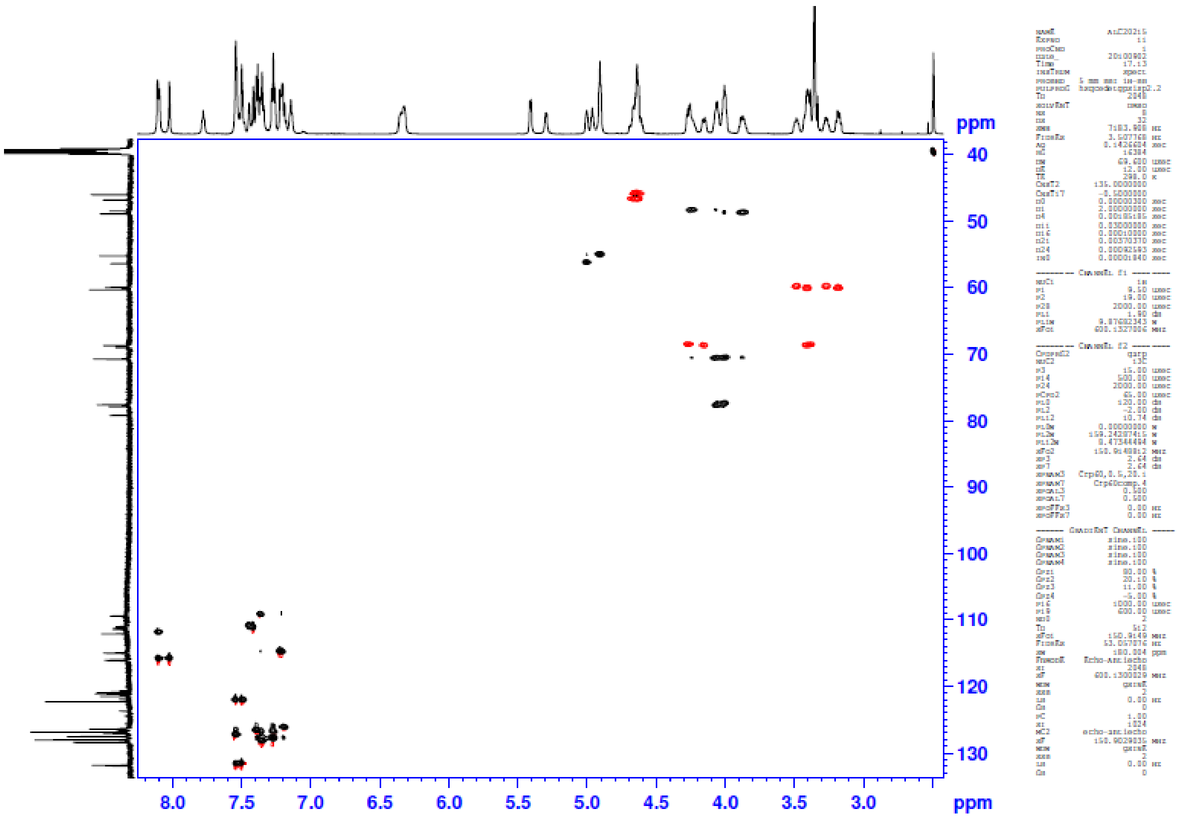


**Supplementary Figure 14**: edHSQC NMR spectrum of ACL20215 in DMSO-$d_6$ recorded at 298K.

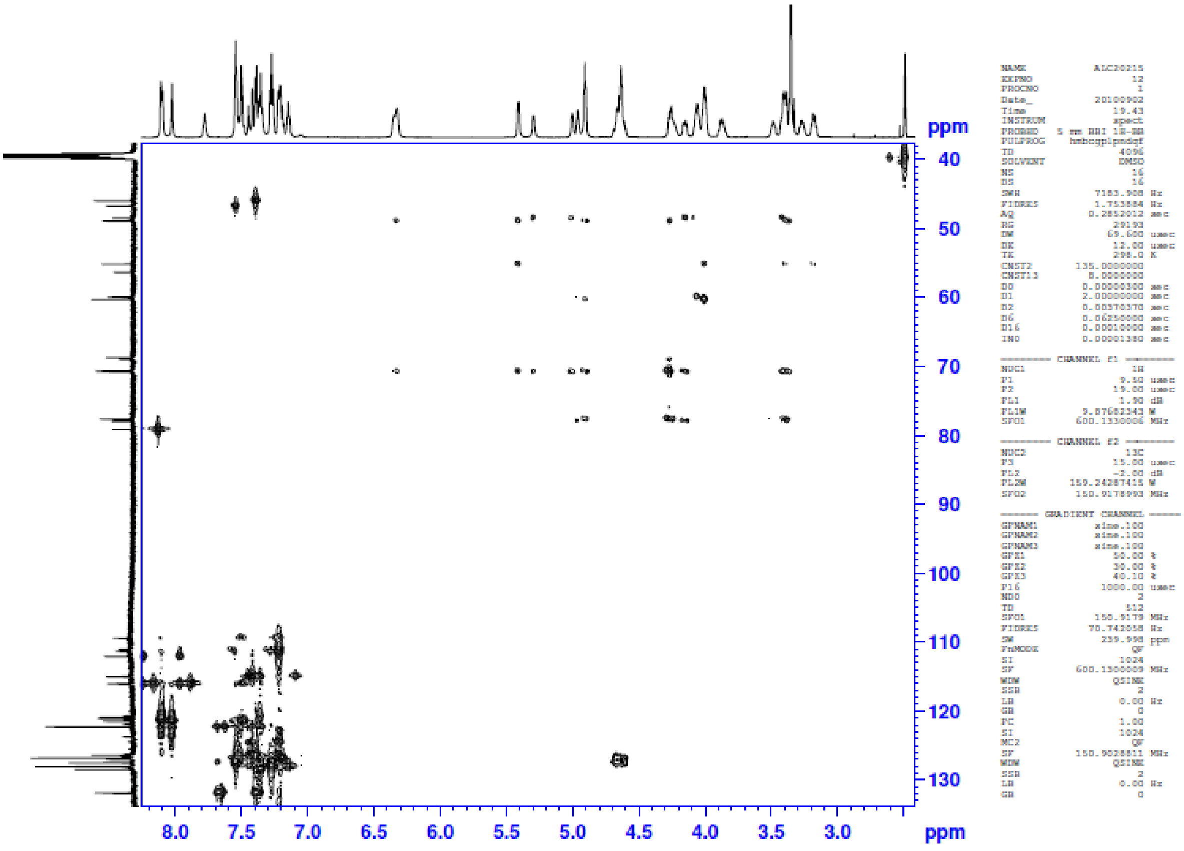


**Supplementary Figure 15:** HMBC NMR spectrum of ACL20215 in DMSO-$d_6$ recorded at 298K.

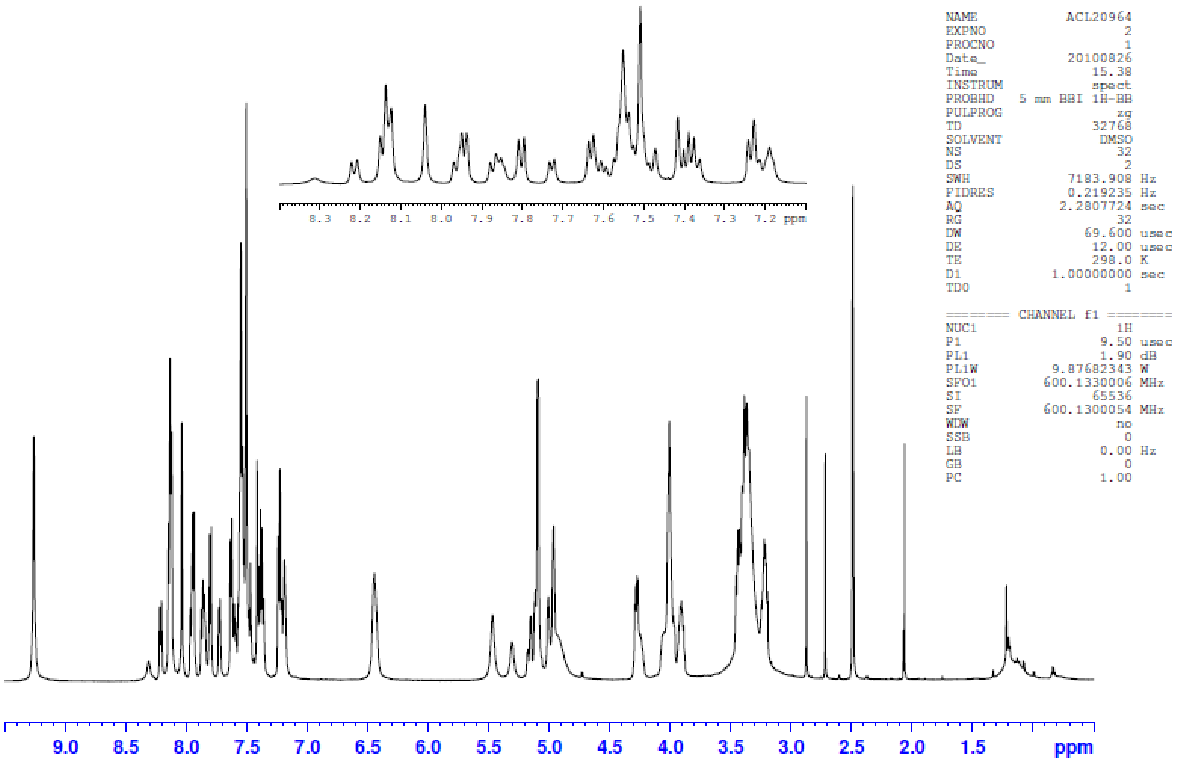


**Supplementary Figure 16:** $^{1}$H NMR spectrum of ACL20964 in DMSO-$d_6$ recorded at 298K.

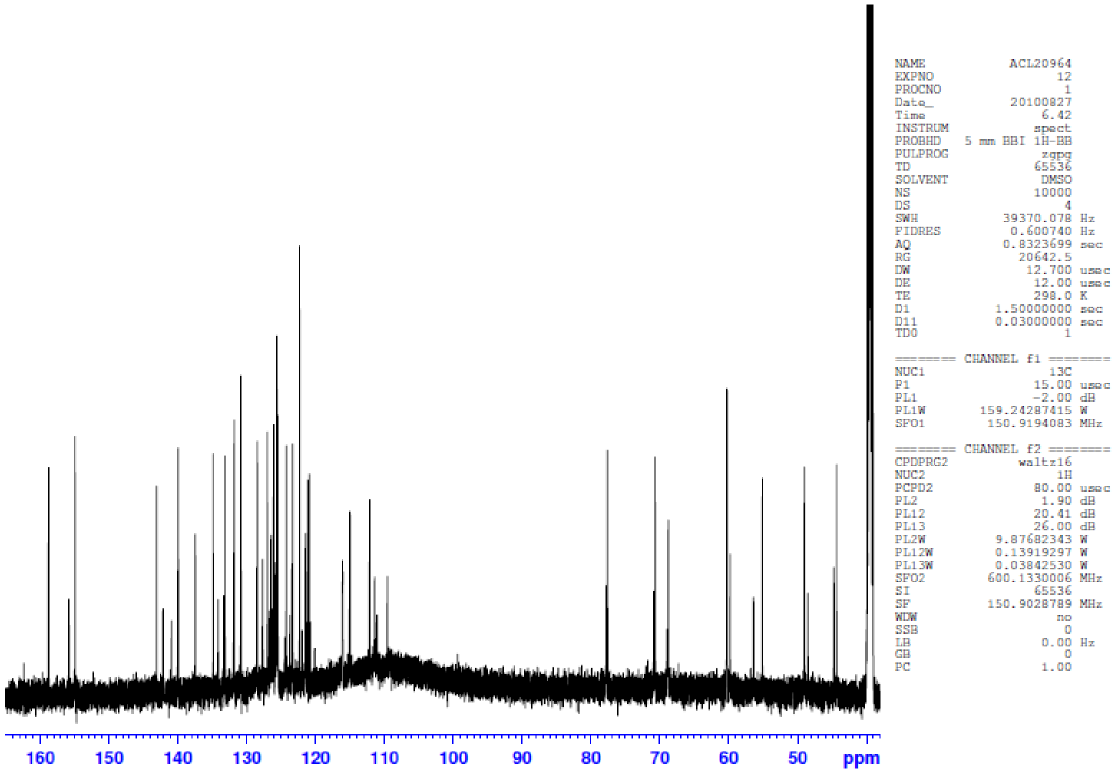


**Supplementary Figure 17:** $^{13}$C NMR spectrum of ACL20964 in DMSO-$d_6$ recorded at 298K.

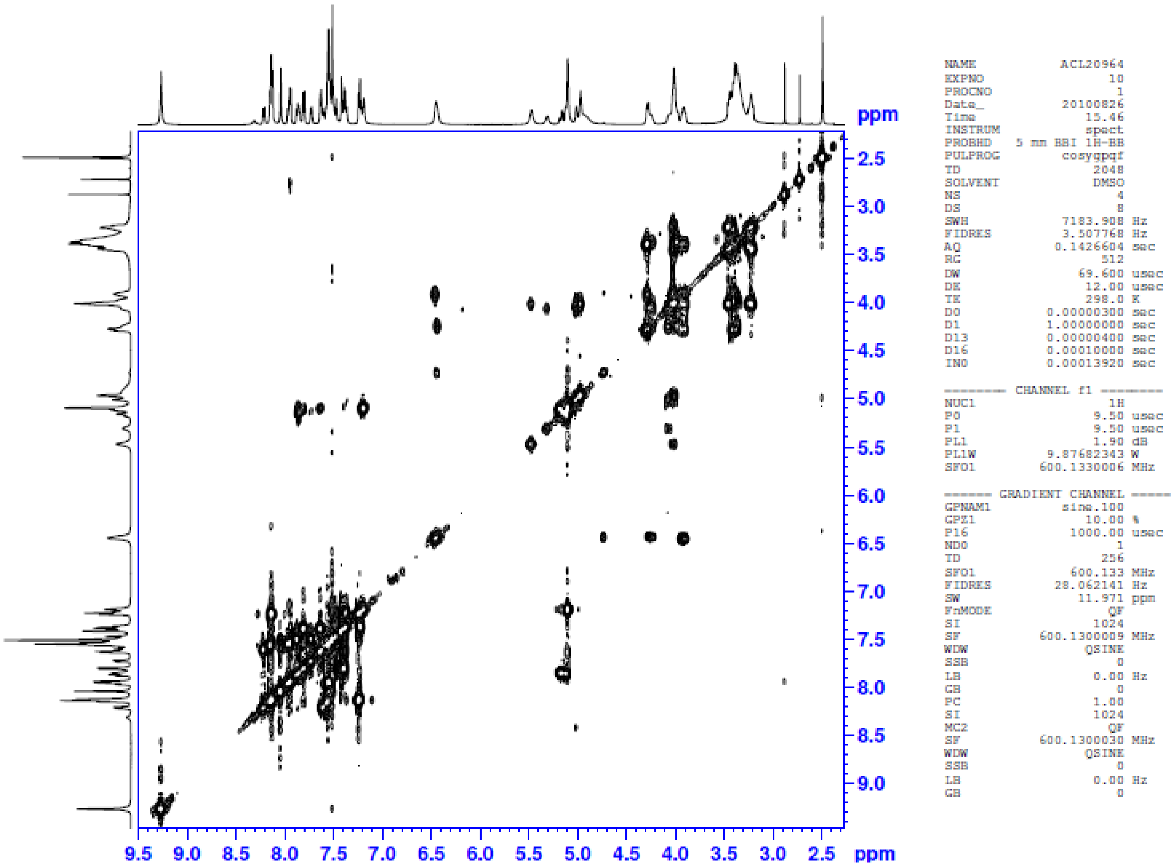


**Supplementary Figure 18:** COSY NMR spectrum of ACL20964 in DMSO-$d_6$ recorded at 298K.

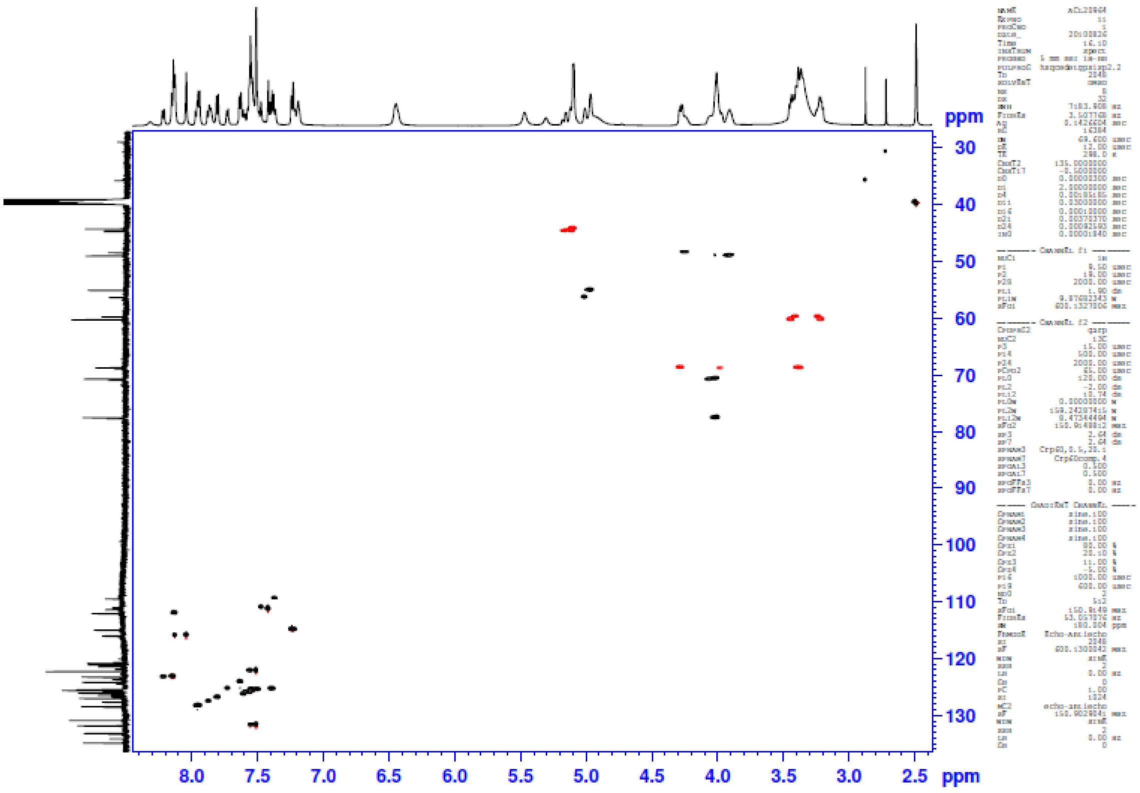


**Supplementary Figure 19:** edHSQC NMR spectrum of ACL20964 in DMSO-$d_6$ recorded at 298K.

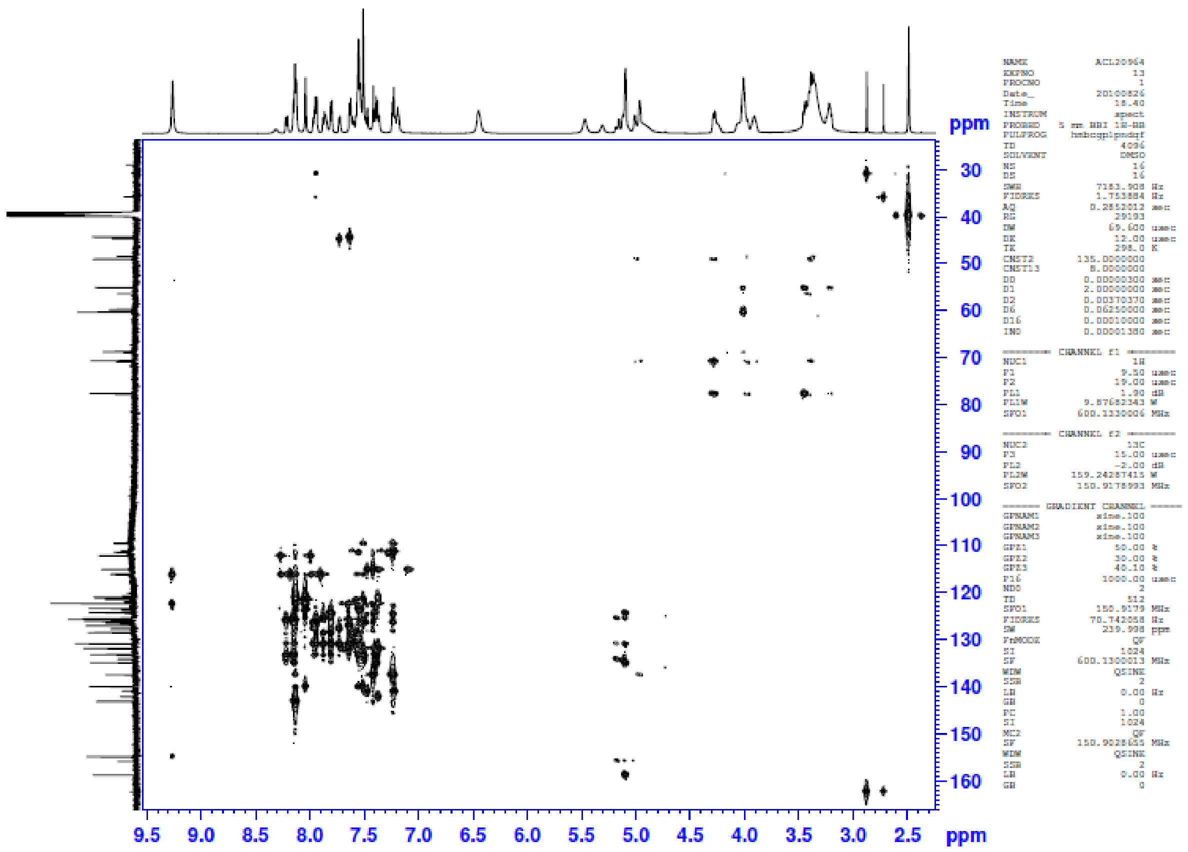


**Supplementary Figure 20:** HMBC NMR of ACL20964 in DMSO-$d_6$ recorded at 298K.

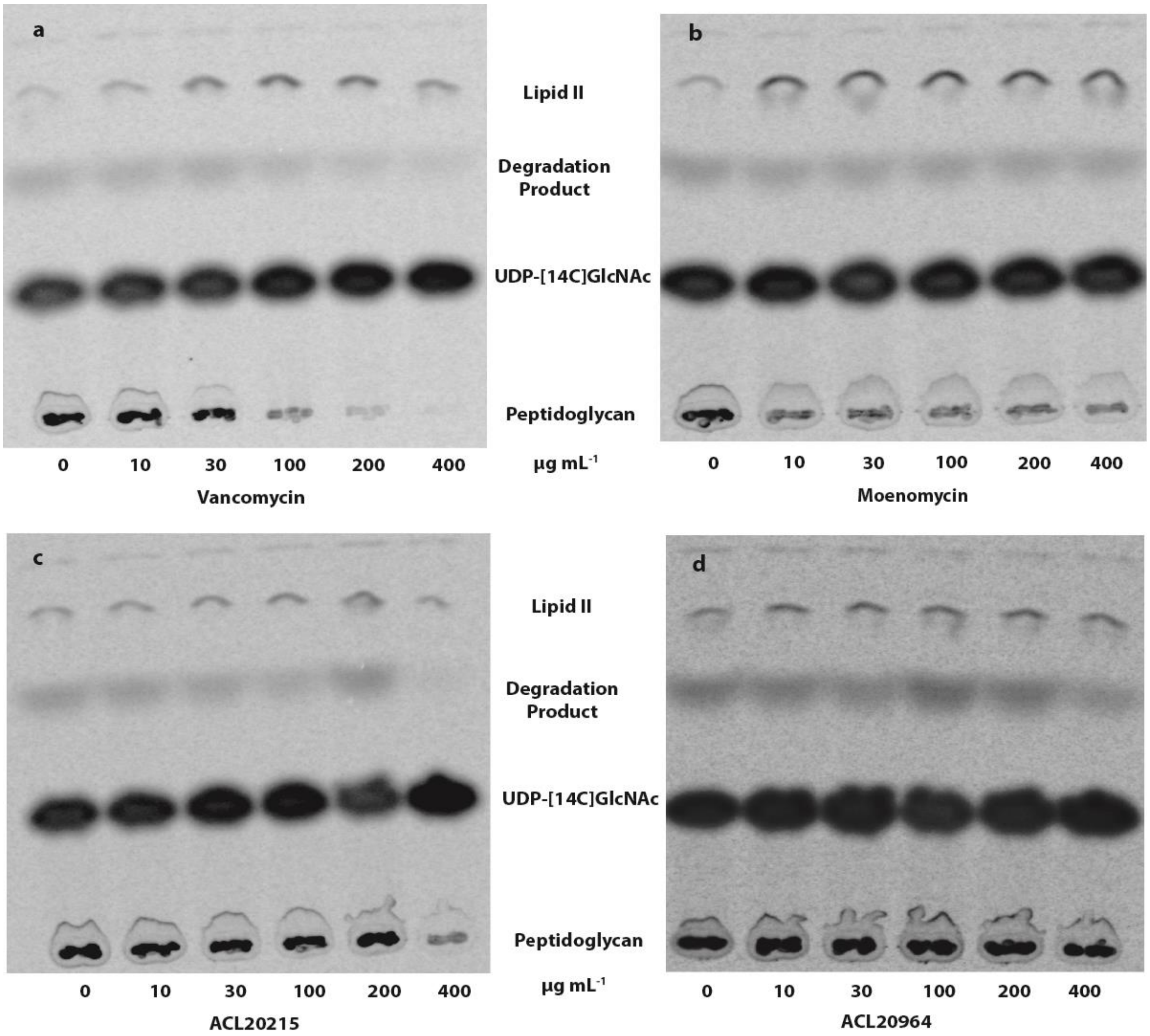


**Supplementary Figure 21:** Inhibition of peptidoglycan synthesis.
TLC separation of *in vitro* bacterial peptidoglycan biosynthesis reaction mixtures in the presence of (a) Vancomycin, (b) moenomycin, (c) ACL20125 or (d) ACL20964.

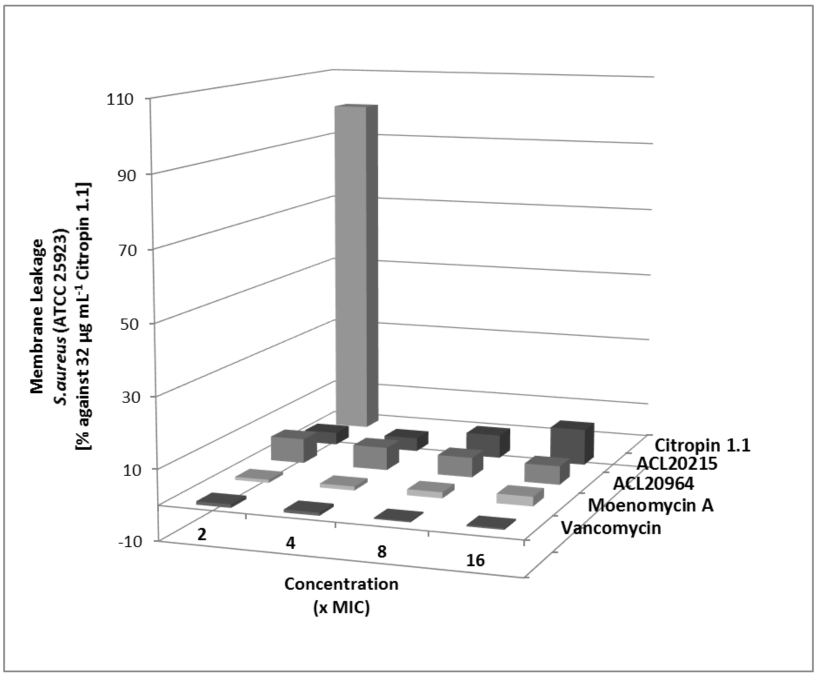


**Supplementary Figure 22**: Cytoplasmic membrane disruption of *S. aureus* (ATCC25923).
Membrane depolarization caused by different antibiotics compared to 32 µg mL$^{-1}$ citropin 1.1 (100%).

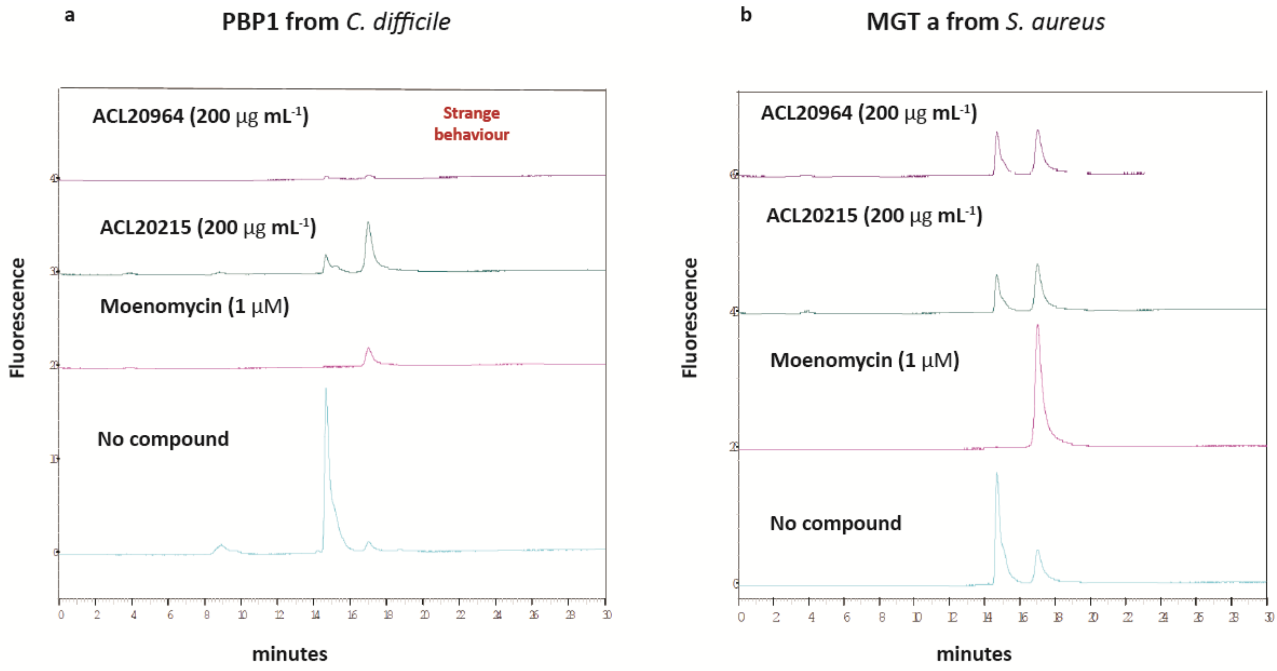


**Supplementary Figure 23**: Inhibition of glycosyltransferases.
The inhibition of two glycosyltransferases, (a) PBP1 from *C. difficile* and (b) MGT-A *S. aureus* for moenomycin A (1 µM), ACL20215 (200 µg mL$^{-1}$) or ACL20964 (200 µg mL$^{-1}$).

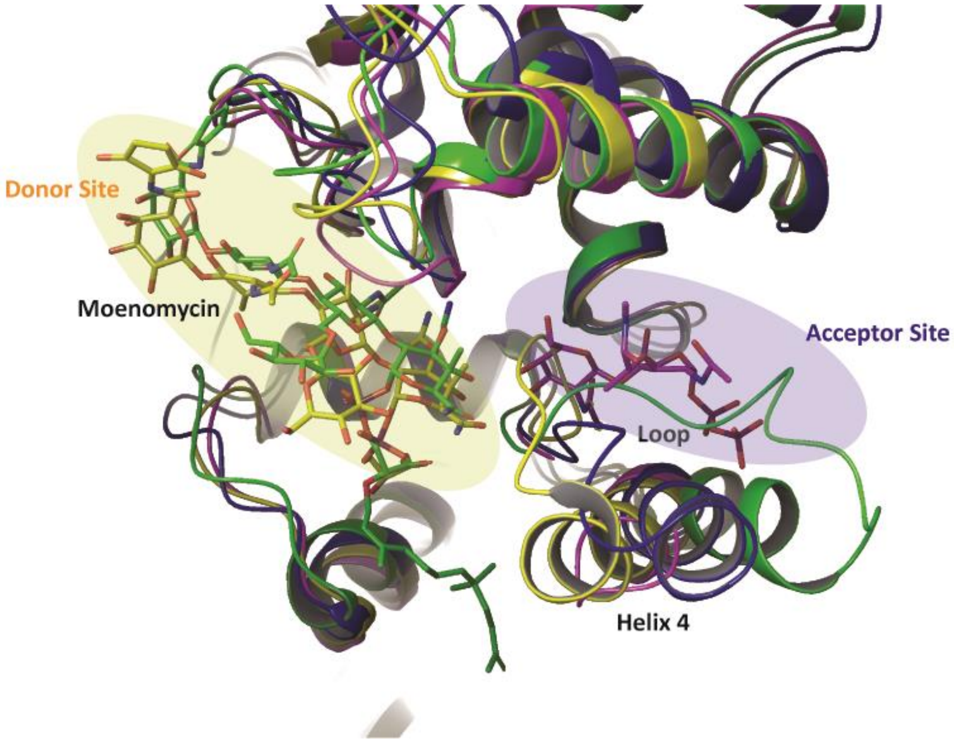


**Supplementary Figure 24**: Binding site loop of different structures of MGT from *S. aureus.* Green: 3HZS[1] MGT(E100Q) with moenomycin; yellow: 3VMR[2] MGT with moenomycin, pink: 3VMT[2] MGT with Lipid II analogue and blue: MGT homology modelled on PGT of *A. aeolicus* 3NB6 [7].

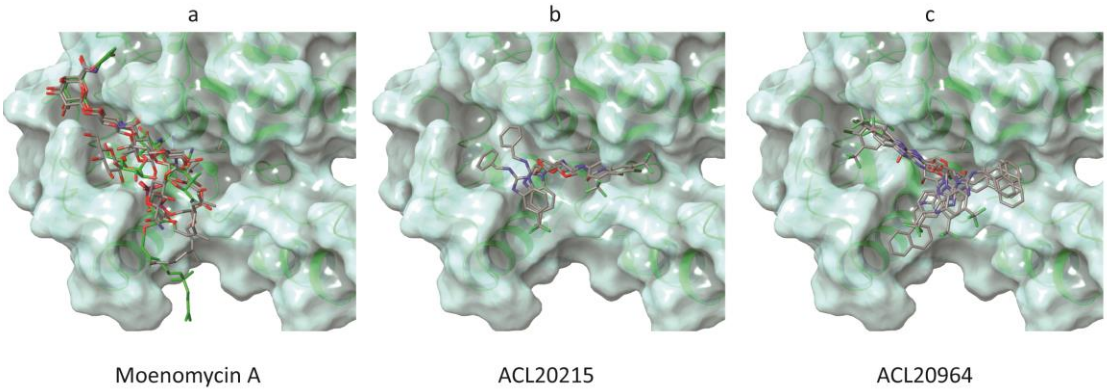


**Supplementary Figure 25**: Virtual docking studies of ACL202158 and ACL20964 in the 3HZSm model. GT domain structures of *A. aeolicus* TG domain (dark grey, *Pdb*: 3NB6 [7]) and *S. aureus* TG domain (light grey, *Pdb*: 2OLV [3]), with (**a**) the crystal structure of moenomycin A (*Pdb*: 2OLV [3]), (**b**) docked structures of ACL19378 (disaccharide) and (**c**) ACL20964 (monosaccharide). The dotted line is illustrating the suggested membrane interface[3].

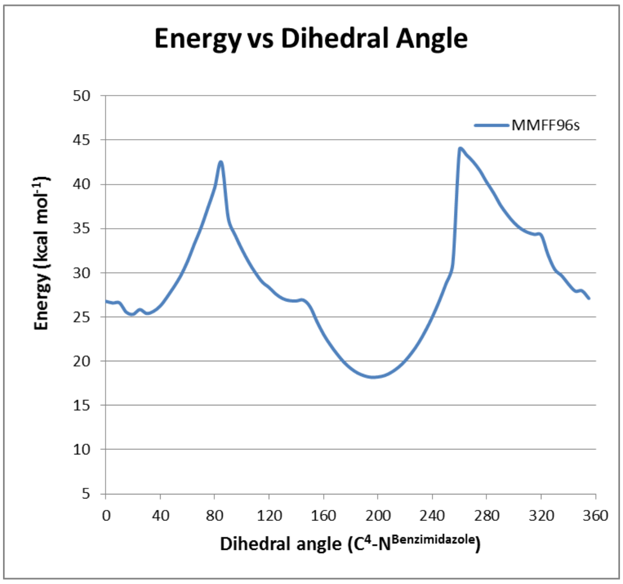


**Supplementary Figure 26**: Dihedral angle of benzimidazole-carbohydrate bond in ACL20215

## Supplementary Tables

**Supplementary Table 1**: MIC data for substituted aminobenzimidazole (chemotype M3)

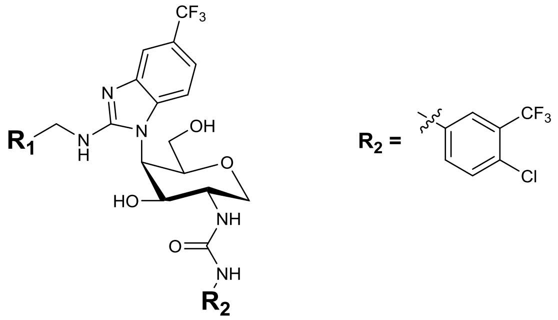


| | | MIC (μg mL$^{-1}$) | | | | | |
|---|---|---|---|---|---|---|---|
| | | *S.aureus* | | *E. faecium* | | *E. faecalis* | *E.coli* |
| **Compound** | **R$_1$** | MSSA | MRSA | - | VanB | - | - |
| ACL20215 | 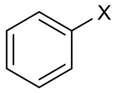 | 4 | 2 | 16 | 16 | 4 | >64 |
| ACL20216 | 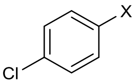 | 4 | 2 | >64 | 4 | | >64 |
| ACL20217 | 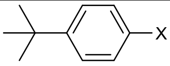 | 8 | 2 | 4 | 2 | 4 | >64 |
| ACL20218 | 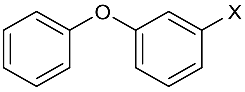 | 4 | 2 | 4 | 2 | 2 | >64 |
| ACL20219 | 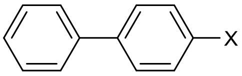 | >128 | - | - | - | - | - |
| ACL20220 | 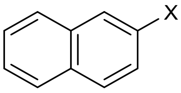 | 1 | 1 | 4 | 4 | 1 | >64 |
| ACL20962 | 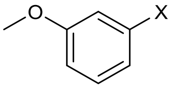 | 16 | 16 | 16 | >64 | 32 | >64 |
| ACL20963 | 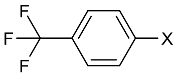 | 4 | 8 | 4 | 8 | 8 | 16 |
| ACL20964 | 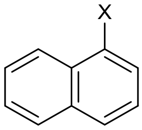 | 4 | 8 | 2 | 2 | 8 | 32 |
| ACL20965 | 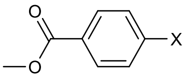 | 8 | 8 | 8 | 8 | 8 | 16 |
| ACL20966 | 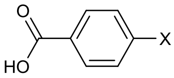 | >128 | - | - | - | - | - |
| ACL20967 | 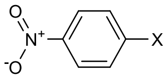 | 8 | 16 | 8 | 32 | 8 | 16 |

**Supplementary Table 2**: Inhibition of lipid II and peptidoglycan

Integrated density values for peptidoglycan and lipid II accumulation at different compound concentration.

| Compound | Concentration ($\mu g\ ml^{-1}$) | Inhibit of PG (%) | Accumulation of lipid II (%) |
|---|---|---|---|
| Vancomycin | 10 | 12.8 ± 2.0 | 36.6 ± 6.2 |
| | 30 | 15.0 ± 3.5 | 106.8 ± 33.4 |
| | 100 | 21.3 ± 1.4 | 140.6 ± 30.8 |
| | 200 | 36.0 ± 2.8 | 151.5 ± 23.1 |
| Moenomycin | 10 | 11.0 ± 0.5 | 97.5 ± 23.9 |
| | 30 | 19.5 ± 3.6 | 103.8 ± 22.2 |
| | 100 | 29.0 ± 1.0 | 126.4 ± 36.2 |
| | 200 | 42.3 ± 0.1 | 142.0 ± 36.9 |
| ACL20215 | 10 | 0.5 ± 6.7 | -3.4 ± 12.7 |
| | 30 | 7.9 ± 0.9 | 17.7 ± 7.1 |
| | 100 | 17.0 ± 1.0 | 34.9 ± 0.6 |
| | 200 | 24.6 ± 5.9 | 47.1 ± 2.8 |
| ACL20964 | 10 | 7.3 ± 0.8 | 24.1 ± 15.0 |
| | 30 | 13.0 ± 2.0 | 31.4 ± 15.9 |
| | 100 | 22.6 ± 1.4 | 45.1 ± 14.9 |
| | 200 | 33.5 ± 3.4 | 58.1 ± 16.8 |

**Supplementary Table 3**: Crystal structures of GT domains

| Bacteria | GT | PDB | Res (Å) | Ligand | Unresolved loop residues (length) |
|---|---|---|---|---|---|
| *Staphylococcus aureus* | MGT | 3HZS[1] | 2.10 | Moenomycin | - |
| | | 3VMQ[2] | 2.52 | - | I123 – V128 (6) |
| | | 3VMR[2] | 3.69 | Moenomycin | V128 – Q219 (2) |
| | | 3VMS[2] | 3.20 | NBD-Lipid II* | I123 – Q219 (7) |
| | | 3VMT[2] | 2.30 | Lipid II analogue | I123 – Q219 (7) |
| *Staphylococcus aureus* | PBP2 | 2OLU[3] | 2.90 | - | K127 – G139 (13) |
| | | 2OLV[3] | 2.80 | Moenomycin | L137 – E144 (8) |
| | | 3DWK[4] | 3.10 | - | - |
| *Escherichia coli* | PBP1b | 3FWL[5] | 3.09 | - | G249 – T267 (19) |
| | | 3VMA[5] | 2.16 | Moenomycin | A251– A265 (15) |
| *Aquifex aeolicus* | PGT | 2OQO[6] | 2.10 | - | Y106 – I111 (6) |
| | | 3D3H[9] | 2.31 | Neryl moenomycin | N105 – Q113 (9) |
| | | 3NB7[7] | 2.65 | Decarb. Neryl Moenomycin[a)] | N105 – V112 (8) |
| | | 3NB6[7] | 2.70 | $MePO_4$Neryl Moenomycin* | - |

[a)] Electron density and low occupancy of ligand reported but no ligand structure modelled.

**Supplementary Table 4**: PK data for ACL20215 and ACL20964.

| **Compound** | **ACL20215** | **ACL20964** |
|---|---|---|
| Administration | IV | IV |
| Measured dose (mg $kg^{-1}$) | 3.5 | 3.5 |
| Apparent $t_{½}$ (h) | 27.2 | 33.8 |
| Plasma $CL_{total}$ (mL $min^{-1}$ $kg^{-1}$) | 42.1 | 17.9 |
| Blood $CL_{total}$ (mL $min^{-1}$ $kg^{-1}$) | 48.9 | 21.5 |
| Blood-to-Plasma ratio | 0.86 | 0.83 |
| $V_D$ (L $kg^{-1}$) | 97.2 | 53.0 |
| % Dose in urine | <LLQ | <LLQ |

<LLQ: Below the lower limit of quantification. $V_D$: Volume of distribution during elimination phase after IV administration. $CL_{total}$: Total clearance after IV administration.

**Supplementary Table 5**: CFU counts for *S. aureus* infected glands after treatment with different doses of ACL20215 and ACL20964.

| **Compound** | **Dose (µg $gland^{-1}$)** | **Glands (#)** | **CFU/g gland ($log_{10}$)** | **Glands Cleared (%)** |
|---|---|---|---|---|
| **ACL20215** | 0 | 10 | 8.74 ± 0.99 | 0 |
| | 200 | 10 | 8.36 ± 1.05 | 0 |
| | 400 | 8 | 7.77 ± 0.52 | 0 |
| | 800 | 10 | 6.60 ± 1.42 | 0 |
| | 1000 | 8 | 5.72 ± 0.63 | 0 |
| **ACL20964** | 0 | 9 | 8.20 ± 0.81 | 0 |
| | 200 | 8 | 7.81 ± 0.45 | 0 |
| | 400 | 8 | 6.76 ± 0.52 | 0 |
| | 800 | 10 | 3.74 ± 1.06 | 10 |
| | 1000 | 8 | 1.95 ± 0.51 | 75 |

**Supplementary Table 6**: The equations for $ED_{2logCFU}$ and $ED_{4logCFU}$ calculation, with best $R^2$

| | **Equation** | **$R^2$** | **Equation type** |
|---|---|---|---|
| **ACL20215** | y = -0.003x + 8.8766 | 0.9865 | linear |
| **ACL20964** | y = -5.10-6 $x^2$ - 0.0013x + 8.2342 | 0.9991 | polynomial |

**Supplementary Note 1**

**Structural variation of GT domains**

Several crystal structures of GT domains have been reported for Gram-positive (MGT[1,2] and PBP2[3,4] from *S. aureus*) and Gram-negative bacteria (PBP1[5] from *E. coli* and PGT[6,7] from *Aquifex aeolicus*), showing a high structural similarity between the difference species. One main feature of the structures is its binding site loop (MGT *S. aureus* $Phe_{120}$-$Gly_{130}$; PBP2 *S. aureus* $Gly_{134}$-$Gly_{145}$)[8] located between the donor binding site, occupied by moenomycin, and the acceptor binding site occupied by the incoming Lipid II molecule. This binding site loop is highly flexibly and partly disordered in most of the crystal structures (see Supplementary Table 3), and even when the loop is resolved it can occupy, together with helix 4, different conformations, either separating the donor from the acceptor sites or opening a groove between the sites. For example MGT from *S. aureus* with moenomycin has been reported twice (*Pdb*: 3HZS[1] and *Pdb*:3VMR[2]) with significant differences in the conformation of helix 4 and the binding site loop (see green and yellow structure in Supplementary Figure 25) The only differences are that one has been crystallized in a trigonal and the other in an orthorhombic space group, and the former is an active site mutant E100Q, however both structures show moenomycin in the same binding orientation.

**Virtual docking**

The crystal structures with moenomycin A locates moenomycin in the donor binding site constituting the growing polysaccharide chain [3,8], with the *N*-Ac group of ring F close to the catalytic site residue Glu100 (numbering by MGT from *S. aureus*). The virtual docking experiments using the ‘best’ receptor model 3HZSm (MGT from *S. aureus*), shows similar overall binding for both ACL20215 and ACL20964**,** but with different orientations of their benzimidazole groups. ACL20215 has its benzimidazole group close to site occupied by ring F of moenomycin, with the benzyl group along the donor site (occupied by ring E), pointing the trifluoro benzyl group towards the acceptor binding site. For ACL20964, the benzimidazole group is still close to site occupied by ring F of moenomycin, but the naphthyl group is orientated towards the acceptor site, while the trifluoro benzyl group towards the donor site. Even though the orientation of both inhibitor is switched around, their binding orientation is dominated by the three hydrophobic groups: trifluoro benzyl group, benzimidazole, and benzyl or naphthyl group. One located at the donor site (similar to ring E), one oriented towards the membrane (similar to portion G of moenomycin) and one orientated towards the acceptor site. This last interaction site is not occupied by moenomycin itself.

**Conformation of Inhibitors**

The compounds with benzimidazole (ACL20215 and ACL20964) exist as atropisomer[13], conformational isomers, due to the bulkiness of the benzimidazole group in vicinity to the carbohydrate ring, restricting the rotation of the benzimidazole linkage bond to the carbohydrate scaffold. An energy barrier of around 25 kcal mol$^{-1}$ for this bond which - in relation to other known atropisomers - corresponds to an interconversion rate of a few hours and up to few days[14]. This corroborates our observations that while two isomers could be detected in the NMR experiment, they could not be separated using chromatography.

## Supplementary Methods

### Conformational (rotamer) search of ACL20215

Both monosaccharide compounds showed distinct conformations in the $^1$H-NMR, which is due to the benzimidazole group restricting the free rotation of the $C^4 – N^{Benzimidazole}$ bond. In order to calculate the rotation energy barrier of that bond, ACL20215 was built using Sybyl (Tripos, St.Louis), and the most predominant $^4C_1$ ring conformation for the monosaccharide scaffold. The structure was energy minimized using the MMFF96s force field before using the systematic torsion grid search, which changed the torsion angle in 5 degree increment, and minimized each conformation (MMFF96S force field) while constraining the torsion angle. This results in 75 conformations of ACL20215 with different torsion angles (see Supplementary Fig. 25). While the energy landscape is not optimized it gives a good indication of the upper range of the energy barrier of ~25 kcal mol$^{-1}$ ($E_{Min1}$: 18.19; $E_{Min2}$: 25.31; $E_{Max1}$: 43.96 $E_{Max2}$: 42.46 kcal mol$^{-1}$).

### Hemolysis assay

Human male red blood cells were isolated from fresh whole blood sample and washed with phosphate buffered saline (PBS) 3 times. Red blood cells were diluted 1/200 with PBS for the haemolysis assay. Each compound was assayed at 4 different concentrations in quadruplicate. Control items (100 µM tamoxifen and 1% triton X100) were also assayed in quadruplicate. Background was determined with 1% DMF. The assay was performed for 1 h at 37 °C. The samples were then centrifuged for 10 min at 4°C at 1000 × *g*, the supernatant was recovered and absorption was measured at 415 nm. Results are expressed as the % haemolysis compared to 1% triton X100 after background was subtracted, and fitting dose response curve with Prism (GraphPad) to extract $HC_{50}$ values.

### Bacterial Cytoplasmic Membrane Permeability Assay

The depolarization of the cytoplasmic membrane of *S. aureus* (ATCC25923) caused by the antibiotics was determined using the membrane potential-sensitive cyanine dye $diSC_35$ and using the method described by Wu *et al.*[15] with some modification. Briefly, the overnight subcultures of bacteria were grown in LB broth at 37 °C with shaking to mid-logarithmic phase ($OD_{600}$ = 0.5 to 0.6). Cells were collected by centrifugation at 5,000 × *g* for 10 min, washed once with the buffer (5 mM HEPES, pH 7.2, 5 mM glucose) and resuspended in the same buffer to an $OD_{600}$ of 0.005. The cell suspension was incubated with 0.4 µM $diSC_35$ until there was a stable (approximately 90%) reduction in fluorescence due to $diSC_35$ uptake and quenching in the cells in response to an intact membrane potential. Then

100 mM KCl was added to equilibrate the cytoplasmic and external $K^+$ concentrations. A 90 µL aliquot of cell suspension was placed in an OptiplateTM 96-well white microplate (Perkin Elmer Corp, Australia). The fluorescence intensity was monitored by using a BMG Labtech PolarStar Omega multimode reader fitted with 620-10 and 665-10 excitation and emission filters respectively, at an excitation wavelength of 622 nm and an emission wavelength of 670 nm. After allowing the dye signal to stabilize for about 5 min, 10 µl of 10-fold concentrated tested compounds dissolved in 10% DMSO were added to yield final concentrations at 2-, 4-, 8-, and 16-fold of individual MIC respectively. Changes in fluorescence due to the disruption of the membrane potential gradient in the cytoplasmic membrane were recorded in real-time. A blank with only cell suspension and dye was used to subtract the background. The membrane permeability assay was performed in triplicate (n = 3). The membrane depolarization as measured by the fluorescence recovery ($F_r$) was defined by the equation:

$$F_r = (F_m - F_b) - (F_i - F_b) \quad (1)$$

where $F_m$ is the maximal fluorescence increase after the addition of a compound at a particular concentration; $F_i$ is the initial fluorescence (the fluorescence level of cell suspension just after the addition of compound) and $F_b$ is the fluorescence of blank. Citropin 1.1, which is known to disrupt membrane integrity of bacteria, was used as a positive control (100%) at a concentration of 32 µg $mL^{-1}$ in this study [16-18].

**Inhibition of Lipid II and peptidoglycan biosynthesis**

The cell-free particulate fraction of *Bacillus megaterium* KM (ATCC13632), capable of catalysing the polymerisation of PG from UPD-linked precursors was performed as described previously [19] with some modification. *Bacillus megaterium* was grown in the medium containing tryptone (Difco-Bacto), 1% (w/v); yeast extract (Difco-Bacto), 0.5% (w/v); $K_2HPO_4$, 0.25% (w/v); glucose, 0.5% (w/v); and adjusting the pH to 7.2. Bacteria were harvested when the density had reached to 0.4 mg dry weight $mL^{-1}$ and washed in the buffer 0.05 M Tris-HCl (pH 7.8) containing 10 mM $MgCl_2$ by centrifugation at 6,000 x *g* for 10 min at 4 °C. All subsequent manipulations were carried out as close to 0 °C as possible. The bacteria were resuspended in the same buffer at a density of 50 mg dry weight $ml^{-1}$. Then the bacteria were subjected to three freeze/thaw cycles (dry ice for 5 min followed by RT for 10 min). After the freeze/thaw cycles, bacteria were homogenized by using the glass homogenizer at 4 °C (10 min × 2). The broken cell suspension was centrifuged at 6,000 x *g* for 10 min at 4 °C. The supernatant which contained the unbroken cells and the majority of the cell walls was kept for further centrifugation. The pellet was resuspended in the same buffer and re-centrifuged at 6000 x *g* for 10 min at 4 °C, and the supernatant was kept for further centrifugation. The two supernatants were combined and

centrifuged at 20,500 rpm for 1 h at 4 °C. The pellet, consisting mainly of membrane fragments together with some cell wall material, was washed once with the same buffer and finally resuspended in 0.05 M Tris-HCl (pH 7.8) containing 10 mM $MgCl_2$. Protein concentration was measured by the method of Bradford using crystalline bovine serum albumin (BSA) as standard [20] The reaction mixtures contained, in a final volume of 20 µL, 50 mM Tris-HCl (pH 7.8), 10 mM $MgCl_2$, membrane faction (around 40 to 50 µg protein), 0.4 mM UDP-*N*-acetylmuramyl-pentapeptide (UDP-MurNAc-pentapeptide, The University of Warwick, UK), 6.5 µM [$^{14}$C]UDP-*N*-acetylglucosamine (UDP-[$^{14}$C]GlcNAc, American Radiolabeled Chemicals Inc, 25µci, 11.1 GBq $mmol^{-1}$, 0.1 mci $mL^{-1}$) and either vancomycin hydrochloride (Sigma Aldrich, Australia), moenomycin A (Sigma Aldrich, Australia), or compounds at the final concentrations of 0, 10, 30, 100, 200 µg $mL^{-1}$ in 2% DMSO. The reaction mixtures were incubated at RT for 3 h and placed in a boiling water bath for 3 min to inactivate enzymes to prevent degradation of lipid II. Aliquots (5 µL) of the samples were separated by TLC on silica gel plates (TLC Silica gel 60 F254, 20 × 20cm, Merck KGaA, Germany) for 2 h in isobutyric acid /1M $NH_4OH$ (5/3, v/v). After the aliquots were separated, the plates were dried and exposed to phosphorimaging screen (GE Healthcare, Australia), for 1 week and scanned by Typhoon 8600 (GE Healthcare, Australia). Integrated Density Value (IDV) of each band on silica gel was analysed by the software AlphaEase FC (AlphaImager 2200). The changes of peptidoglycan and lipid II were calculated as percent from the IDV values of the control (without any antibiotic or compound).

Crude cell membranes were prepared from *Bacillus megaterium* KM, solubilized, and supplied with UDP-[$^{14}$C]GlcNAc and an appropriate UDP-MurNAc-pentapeptide. The samples were treated with various antibiotics in different concentrations prior to the addition of UDP-[$^{14}$C]GlcNAc. Following incubation and inactivation by boiling, 5 µl aliquots of samples were separated by TLC on silica gel plates and the plates were dried and auto-radiographed. TLC autoradiogram was shown: (a) vancomycin; (b) moenomycin; (c) ACL20215 and (d) ACL20964. The experiment was done in triplicate (n = 3).

**Glycosyltransferase activity by HPLC**

A solution containing NBD-lipid II (4 µM) in buffer (50 mM Tris, pH 8.0, 10 mM $CaCl_2$, 0.085% Decyl PEG50, 10% DMSO, 15% MeOH) containing 10 µg $\mu L^{-1}$ *N*-acetylmuramidase was mixed at 1000 rpm at 25 °C. The reaction was initiated by addition of glycosyltransferease (2.5 µg $mL^{-1}$) and terminated by addition of 100 µM moenomycin A in 1/10 volume. The reaction mixtures were then analysed by a HPLC system (Hitachi) equipped with an anion-exchange column (5 µm, 4.6 × 250 mm, SAX1, Supelco Co.) using a linear gradient of $NH_4OAc$/MeOH (20 mM to 1 M) at a flow rate of 1 mL $min^{-1}$. The signals were detected with a fluorometer at $\lambda_{ex}$ = 355 nm and $\lambda_{em}$ = 460 nm.

The results with PBP1 from *C. difficile* showed some inhibition for ACL20215, while the experiment with ACL20964 displays no detection of either the substrate (NBD-lipid II) or the product of the glysyltransferase reaction (marked in Supplementary Figure 23 with 'Strange behaviour'). However the experiment with MGT A from *S. aureus* shows some inhibitory effect for both the compounds. These preliminary results were used to optimise the experiment to extend it to a dose response assay.

***In vitro* metabolic stability studies**

Compounds (1 µM) were incubated at 37 °C with human or mouse liver microsomes. The reaction was initiated by the addition of an NADPH-regenerating system and quenched at various time points over the incubation period by the addition of acetonitrile. Additional samples co-activated by NADP and UDPGA (the co-factor for glucuronidation), were included in the incubation for the qualitative assessment of the potential for glucuronide formation. The relative loss of parent compound and formation of metabolic products was determined by LC/MS using the Q-TOF instrument. The studies were conducted by a contract research organization (Centre for Drug Candidate Organization, Monash University, Melbourne, Australia).

***In vivo* efficacy and PK studies**

The *in vivo* pharmacokinetics of the compounds were studies in overnight-fasted male Sprague Dawley rats weighing 328-334 g. Rats had access to water *ad libitum* throughout the pre- and post-dose sampling period, and access to food was re-instated 4 hours post-dose. Compounds were administrated IV as a 10 minute constant rate infusion (1.0 mL per rat, n=2 rats). Samples of arterial blood and total urine were collected up to 48 h post-dose. Arterial blood was collected directly into borosilicate vials (at 4 °C) containing heparin, Complete (a protease inhibitor cocktail), potassium fluoride and EDTA to minimize potential for *ex vivo* degradation. Once collected, blood samples were centrifuged, supernatant plasma was removed, and plasma concentration of compounds were determined by LCMS (LLQ 0.0014 µM). The studies were conducted by a contract research organization (Centre for Drug Candidate Organization, Monash University, Melbourne, Australia).

***In vivo* Maximum Tolerated Dose**

Compound dissolved in 0.05% Tween 80/0.9% NaCl were each administered IP at doses of 20, 60 and 100 mg $kg^{-1}$ to groups of 2 CD-1 (*Crl.*) derived male mice weighing 22 ± 2 g. The animals were observed for presence of acute toxic symptoms (mortality, convulsions, tremors, muscle relaxation, sedation, etc.) and autonomic effects (diarrhea, salivation, lacrimation, vasodilation, piloerection, etc.) during

the first 30 min post-dosing. Any occurrence of mortality was noted at 3, 24, 48 and 72 h after treatment.

For the IV studies, compounds were dissolved in 10% DMSO/0.1 M HCl/1% Tween 80/5% Glucose were prepared at a dose of 4 mg $kg^{-1}$ and were administered intravenously once to groups of 3 CD-1 (*Crl.*) derived male mice weighing 22 ± 2 g, and monitored and analyzed similar to the IP study during the first 5 min post dosing (IV). In addition, Group 3 and Group 5 were administered intravenously with compounds at 4 mg $kg^{-1}$ for 2 times (T=0 and T=170 minutes) and the autonomic signs and neurological changes were observed during the first 5 min after the first and the second dose. Mortality was also observed at the subsequent 30 min, 1, 24, 48 and 72 h after test substances treatment. Both studies were conducted by a contract research organization (MDS Pharma Services, Taipei, Taiwan).

***In vivo* Septicaemia mouse model**

Groups of 10 male CD-1 (*Crl.*) derived mice weighing 22 ± 2 g were used. The mice were inoculated intraperitoneally (IP) with a $LD_{90-100}$ of *Staphylococcus aureus* (Smith) ($3.7 \times 10^5$ CFU $mouse^{-1}$) in 0.5 ml of brain-heart infusion broth containing 5% mucin. Compounds were dissolved in 0.05% Tween 80/0.9% NaCl. Vehicle and compounds at 50 and 100 mg $kg^{-1}$ were each administered intraperitoneally 10 and/or 180 minutes after *S. aureus* (Smith) challenge. Ampicillin (0.1 mg $kg^{-1}$, IP, as positive control agent) was administered to test animals at 1 h after the inoculation. Mortality was monitored once daily for 7 days. An increase of survival by 50 percent or more (>50%) relative to the vehicle control group after the bacterial inoculations indicates significant antimicrobial effect.

For the corresponding IV studies compounds at 4 mg $kg^{-1}$ were dissolved in 10% DMSO/0.1 M HCl/1% Tween 80/5% glucose, and compounds were each administered intravenously at 10 min and/or 180 min after *S. aureus* (Smith) challenge.

***In vivo* Mastitis mouse model**

For the infection of the mice *S. aureus* Newbould 305 (ATCC 29740) was used , which was originally isolated from a clinical mastitis case [21]. Bacterial preparation was previously described [22]. Briefly, an overnight brain heart infusion (BHI; Oxoid Limited) culture of the bacteria was diluted in sterile phosphate-buffered saline (PBS; Gibco) and quantified by flow cytometry (BD biosciences). Subsequently, 1 ml of a 1000-fold PBS-diluted bacterial suspension was added to a TRUcount tube (BD biosciences), which contained a known number of fluorescent beads. The number of bacteria was then calculated using the following equation:

$$bacteria\ per\ mL = \frac{\#\ bacterial\ count \times beads\ in\ tube \times dilution}{\#\ beads\ counts} \qquad (2)$$

The actual colony forming units (CFU) of the inocula was confirmed by overnight culture of a serial logarithmic dilution on tryptic soy agar (TSA; Oxoid Limited) plates.

Determination of the MIC of compounds for the experimental bovine mastitis isolate *S. aureus* Newbould 305 was performed using the Mueller-Hinton agar dilution assay according to CLSI guidelines [23]. Plates were incubated at 35 °C (+/-2 °C) for 16-20 h in an aerobic atmosphere. In addition the MIC of a random selection of recent bovine mastitis field isolates (10 isolates for each antibiotic) was determined in order to ensure that the sensitivity of the experimental isolate concurred with the sensitivity of a naïve bacterial population encountered in the field.

The procedure for mouse mammary gland infection has been recently described [22]. Briefly, CD-1 lactating mice (Harlan Laboratories Inc.) were utilized 12 to 14 days after birth of the offspring. The pups were weaned 1 to 2 h before bacterial inoculation of the mammary glands. A mixture of oxygen and isoflurane (2-3%) was used for inhalational anaesthesia of the lactating mice. A syringe with 32-gauge blunt needle (Thiebaud biomedical devices) was applied to inoculate both L4 (on the left) and R4 (on the right) glands of the fourth abdominal mammary gland pair with approximately 150 CFU of *S. aureus*. Each orifice was exposed by a small cut at the near end of the teat and 100 µl of the inoculum was injected slowly through the teat canal. The antimicrobial formulation was instilled into the mammary gland of anesthetised mice using the desired dose (µg $gland^{-1}$) at 4h after bacterial inoculation. Immediately thereafter the postoperative analgesic Buprecare (Codifar NV, Belgium) was administered intraperitoneally, *i.e.* at 4 h after bacterial inoculation. The animal experiments were approved by the Ethical Committee of the Faculty of Veterinary Medicine, Ghent University (EC2009/133).

After sacrifice of the mice by cervical dislocation at 14 h post-treatment, mammary glands (two per mouse) were harvested, weighed and homogenized on ice in sterile PBS using a TissueRuptor (QIAGEN Benelux BV). The mammary glands, which are structurally separate, were considered as individual samples. Bacterial CFU counts were obtained after quantification of serial logarithmic dilutions of mammary gland homogenates on TSA plates and transformation of the raw CFU counts into base 10 logarithm values. The detection limit (DL) was 1.7 $log_{10}$ CFU $g^{-1}$ gland weight.

The mean intramammary CFU $g^{-1}$ gland of infected glands from mice treated with different doses of the antimicrobial compound (test group) are compared with the mean CFU $g^{-1}$ gland of infected glands from mice injected with excipient only (negative control group). To evaluate the dose-response relationship for the antimicrobial the $ED_{2logCFU}$, $ED_{4logCFU}$, $PD_{50}$ and $PD_{100}$ values were calculated. The $ED_{2logCFU}$ and $ED_{4logCFU}$ are the concentrations of the antimicrobial needed to reduce the mean CFU $g^{-1}$ gland from the control group by $2log_{10}$ and $4log_{10}$, respectively. The $PD_{50}$ and $PD_{100}$ are the doses of

antimicrobial needed to observe respectively 50% and 100% of cleared mammary glands, i.e. with CFU $g^{-1}$ below the detection limit (DL). The equations that fitted best, i.e. yielded the highest $R^2$, with the dose-response relationship of the antibiotic are summarised in Supplementary Table 6. The $ED_{2log10}$ and $ED_{4log10}$ values were then computed by calculating 'x' from these equations, in which 'y' is replaced by 'z-2' and 'z-4', respectively, with 'z' being the mean final CFU $g^{-1}$ from the negative control group.

**Design and Synthesis of Disaccharide-Based Library**

**Synthesis of Disaccharide 2.** Disaccharide **1** (2.0 g, 1.89 mmol) was suspended in a mixture of *n*-butanol (14 ml) and ethylenediamine (14 ml) (1:1) and heated at 120 °C for 40 min. The solvent was removed *in vacuo* and the crude sugar-amine was purified on a squat column (3-5 % MeOH / DCM) to give a white solid, 1.33 g, 76 %. Rt 6.43, $(M+H)^+$ 926. A solution of the sugar-amine (1.33 g, 1.44 mmol) and 3,4-dichlorophenyl isocyanate (325 mg, 1.73 mmol, 1.2 equiv.) in dry DMF (14 ml) was stirred at room temperature for 3 h. The DMF was removed *in vacuo* by azeotroping with toluene to give the urea **2** as a glassy solid, 2.11 g. Due to a lack of solubility of the product it was not purified (by chromatography) and was used crude. Rt 6.98, $(M+Na)^+$ 1135.

**Synthesis of Disaccharide 3.** A solution of the crude azido-sugar **2** (2.11 g) in DMF (11.5 ml) was treated with MeOH (2.9 ml), a solution of $NH_4Cl$ (770 mg, 14.4 mmol, 10 equiv.) in $H_2O$ (1.6 ml) and activated zinc dust (5.65 g, 86.4 mmol, 60 equiv.). The suspension was stirred at room temperature overnight. Chloroform (15 ml) was added and the suspension was filtered through celite washing with chloroform/DMF (1:1, 60 ml). The combined filtrates were washed with brine (100 ml), dried ($MgSO_4$) and the solvent was removed *in vacuo* to give the sugar-amine as a white solid, 1.57 g, 100 %. Rt 6.88, $(M+H)^+$ 1087. To a solution of 3-([1,1'-biphenyl]-4-yl)-3-((*tert*-butoxycarbonyl)amino)propanoic acid (118 mg, 0.346 mmol, 1.25 equiv.) in dry DMF (0.7 ml) was added a solution of HBTU in dry DMF (0.7 ml, 0.5 M, 0.35 mmol, 1.25 equiv.) followed by diisopropylethylamine (DIPEA) (0.2 ml, 1.15 mmol, 4.2 equiv.). The solution was stirred for 10 min and then a solution of the sugar-amine (300 mg, 0.276 mmol) in dry DMF (2.1 ml) was added. The reaction was stirred at room temperature overnight and then diluted with DCM (15 mL), washed with 10% citric acid (15 ml), saturated $NaHCO_3$ (15 ml) and brine (15 ml), and dried ($MgSO_4$). The solvent was removed *in vacuo* to give disaccharide **3**.

**Synthesis of Disaccharide 4.** To disaccharide **3** (0.276 mmol) was added a solution of TFA (5 %), triethylsilane (15 %) in dry DCM (10 ml). The reaction was stirred at room temperature for 5 h and the solvent was removed *in vacuo*. The resulting disaccharide (0.276 mmol) was dissolved in a solution of saturated ammonia in methanol (10 ml). The reaction was heated at 55 °C for 5 h and the solvent was removed *in vacuo*. A solution of the sugar-amine (0.276 mmol) in THF (5 ml) was treated with di-*tert*-butyl dicarbonate (120 mg, 0.550 mmol, 2 equiv.), water (0.6 ml) and $NaHCO_3$ (until pH 7-8, about 10

equiv.). The reaction was stirred at room temperature for two days, diluted with DCM (25 ml), washed with water (2 x 25 ml), dried ($MgSO_4$) and the solvent was removed *in vacuo* to give disaccharide **4**.

**Synthesis of ACL19378.** To a solution of disaccharide **4** in dry DMF (69 μmol, 0.5 ml, 0.138 M) in the glove box was added a solution of 4-(trifluoromethoxy)phenyl isocyanate (17 mg, 83 μmol, 1.2 equiv.) in dry DMF (0.2 ml, 0.41 M). The reaction was hand shaken and left to sit in the glove box overnight. The solvent was removed *in vacuo*. To the resulting disaccharide (49 μmol) was added a solution of TFA (5%), triethylsilane (15%) in dry DCM (4 ml). The reaction was stirred at room temperature for 2 h and the solvent was removed *in vacuo*. The resulting disaccharide was then dissolved in a solution of saturated ammonia in methanol (3 ml). The reaction mixture was heated at 55 °C for 2 h and the solvent was removed *in vacuo* to give **ACL19378**. The crude product was purified by LCMS on standard C18 column using a water/acetonitrile gradient.

**Analysis data for ACL19378 (NMR and MS)**

MS spectra of ACL19378 were obtained using an AB Sciex TripleTOF® 5600 (ABSCIEX, Canada) equipped with a DuoSpray Ion Source. ACL19378 was infused into the MS system using a Harvard Syringe Pump at 10 µL $min^{-1}$. Data was collected in (+)-ESI-mode from *m/z* 50 to 1000 and product ions from *m/z* 50 to 1000 in high resolution TOF mode (collision energy (CE) 20, ionspray voltage 5300V, declustering potential (DP) 100V, curtain gas flow 25, nebuliser gas 1 (GS1) 12, GS2 to 5, interface heater at 150 $^oC$ and the turbo heater to 450 $^oC$). NMR data were collected in DMSO-$d_6$ using a Bruker Avance 600 MHz spectrometer (Bruker, Germany) at the temperatures noted with spectra calibrated to residual solvent signals ($\delta_H$ 2.49 and $\delta_C$ 39.0).

.

**Supplementary References**


1 Heaslet, H., Shaw, B., Mistry, A. & Miller, A. A. Characterization of the active site of *S. aureus* monofunctional glycosyltransferase (Mtg) by site-directed mutation and structural analysis of the protein complexed with moenomycin. *J. Struct. Biol.* **167**, 129–135 (2009).

2 Huang, C.-Y. *et al.* Crystal structure of *Staphylococcus aureus* transglycosylase in complex with a lipid II analog and elucidation of peptidoglycan synthesis mechanism. *Proc Natl Acad Sci USA* **109**, 6496–6501 (2012).

3 Lovering, A. L., de Castro, L. H., Lim, D. & Strynadka, N. C. J. Structural insight into the transglycosylation step of bacterial cell-wall biosynthesis. *Science* **315**, 1402–1405 (2007).

4 Lovering, A. L., De Castro, L. & Strynadka, N. C. Identification of dynamic structural motifs involved in peptidoglycan glycosyltransfer. *J. Mol. Biol.* **383**, 167–177 (2008).

5 Sung, M.-T. *et al.* Crystal structure of the membrane-bound bifunctional transglycosylase PBP1b from *Escherichia coli*. *Proc. Natl. Acad. Sci. USA* **106**, 8824–8829 (2009).

6 Yuan, Y. *et al.* Crystal structure of a peptidoglycan glycosyltransferase suggests a model for processive glycan chain synthesis. *Proc Natl Acad Sci USA* **104**, 5348–5353 (2007).

7 Fuse, S. *et al.* Functional and structural analysis of a key region of the cell wall inhibitor moenomycin. *ACS Chem. Biol.* **5**, 701–711 (2010).

8 Zuegg, J. & Meutermans, W. Crystal structures of the PBP2 glycosyltransferase domain: new opportunities for antibacterial drug design. *ChemMedChem* **2**, 1403–1404 (2007).

9 Yuan, Y. *et al.* Structural analysis of the contacts anchoring moenomycin to peptidoglycan glycosyltransferases and implications for antibiotic design. *ACS Chem. Biol.* **3**, 429–436 (2008).

10 Friesner, R. A. *et al.* Glide: a new approach for rapid, accurate docking and scoring. 1. Method and assessment of docking accuracy. *J. Med. Chem.* **47**, 1739–1749 (2004).

11 Sherman, W., Beard, H. S. & Farid, R. Use of an induced fit receptor structure in virtual screening. *Chem. Biol. Drug Des.* **67**, 83–84 (2006).

12 Sherman, W., Day, T., Jacobson, M. P., Friesner, R. A. & Farid, R. Novel procedure for modeling ligand/receptor induced fit effects. *J. Med. Chem.* **49**, 534–553 (2006).

13 Oki, M. in *Top. Stereochem.* Vol. 14 (eds N. L. Allinger, N. L. Eliel, & S. H. Wilen) 1–81 (John Wiley & Sons, 1983).

14 Laplante, S. R. *et al.* Assessing atropisomer axial chirality in drug discovery and development. *J. Med. Chem.* **54**, 7005–7022 (2011).

15 Wu, M., Maier, E., Benz, R. & Hancock, R. E. Mechanism of interaction of different classes of cationic antimicrobial peptides with planar bilayers and with the cytoplasmic membrane of *Escherichia coli*. *Biochemistry* **38**, 7235–7242 (1999).

16 Chia, C. S., Gong, Y., Bowie, J. H., Zuegg, J. & Cooper, M. A. Membrane binding and perturbation studies of the antimicrobial peptides caerin, citropin, and maculatin. *Biopolymers* **96**, 147–157 (2011).

17 Giacometti, A. *et al.* *In vitro* activity and killing effect of citropin 1.1 against gram-positive pathogens causing skin and soft tissue infections. *Antimicrob. Agents Chemother.* **49**, 2507–2509 (2005).

18 Wegener, K. L. *et al.* Host defence peptides from the skin glands of the Australian blue mountains tree-frog *Litoria citropa*. Solution structure of the antibacterial peptide citropin 1.1. *Eur. J. Biochem.* **265**, 627–637 (1999).

19 Reynolds, P. E. Peptidoglycan synthesis in bacilli. I. Effect of temperature on the in vitro system from *Bacillus megaterium* and *Bacillus stearothermophilus*. *Biochim. Biophys. Acta* **237**, 239–254 (1971).

20 Bradford, M. M. A rapid and sensitive method for the quantitation of microgram quantities of protein utilizing the principle of protein-dye binding. *Anal. Biochem.* **72**, 248–254 (1976).

21 Prasad, L. B. & Newbould, F. H. Inoculation of the bovine teat duct with *Staphylococcus aureus*: the relationship of teat duct length, milk yield and milking rate to development of intramammary infection. *Can. Vet. J.* **9**, 107–115 (1968).
22 Demon, D. *et al.* The intramammary efficacy of first generation cephalosporins against *Staphylococcus aureus* mastitis in mice. *Vet. Microbiol.* **160**, 141–150 (2012).
23 Clinical and Laboratory Standards Institute. Methods for dilution antimicrobial susceptibility tests for bacteria that grow aerobically; approved standard, 7th ed, M7-A7. CLSI, Wayne, PA (2006).
